# A subcellularly targeted photocaged inhibitor for mitochondrial carbonic anhydrase V†

**DOI:** 10.1039/d5dt01161b

**Published:** 2025-07-04

**Authors:** Noushaba Nusrat Mafy, Kanchan Aggarwal, Sky Price, Dorothea B. Hudson, Elva Ye, Divya Kolli, Emily L. Que

**Affiliations:** a Department of Chemistry, The University of Texas at Austin 105 E. 24th St Austin TX 78712 USA emilyque@cm.utexas.edu

## Abstract

We report the first photoactivatable inhibitor specifically targeting mitochondrial CA-V, a promising anti-obesity target. It features a sulfonamide metal-binding group caged with a photoremovable coumarin and guided to mitochondria *via* a pyridinium moiety. Light exposure removes the cage, enabling precise spatial and temporal inhibition of mitochondrial CA-V activity.

## Abstract 2

Selective targeting of mitochondrial carbonic anhydrases using subcellular directing and photoresponsive protecting groups to mask zinc active site coordinating ligand.

## Graphical abstract

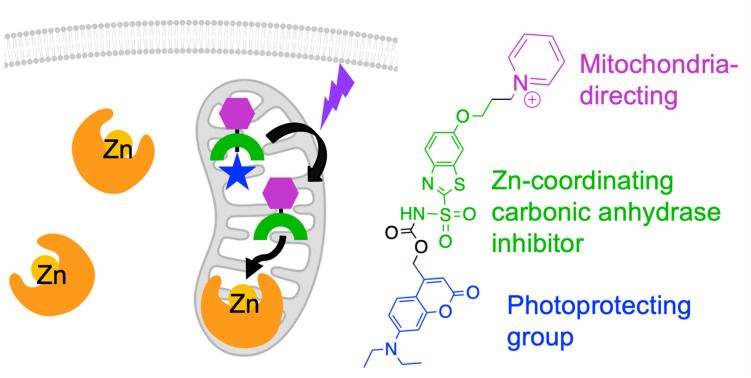

Carbonic anhydrase (CA) is a zinc-dependent metalloenzyme that catalyzes the reversible hydration of carbon dioxide and bicarbonate.^1^ There are 15 known CA isoforms; of these, CA-II and CA-IX have been most extensively studied for inhibitor development.^2,3^ However, mitochondrial isoforms CA-VA and CA-VB, which show 66% sequence similarity, are relatively less explored.^4,5^ CA-VA is most abundant in the liver^6^ and is involved in crucial biosynthetic processes including ureagenesis, lipogenesis, and others.^7–10^ Dysregulation of CA-VA is linked to obesity, diabetes, hyperammonemia, growth deficiencies, and brain-related oxidative stress illnesses.^6,11–17^ CA-VB has a broad tissue distribution, however much less is known about its biological function.^6,18^ Despite the health relevance of CA-VA and CA-VB, limited research has been conducted compared to the more ubiquitous CA-II and CA-IX due to the challenge of selectively targeting CA-V isoforms within cells and organisms.

Current methods for studying CA-V isoforms can be categorized into three main approaches. First, knockout (KO) mouse models can provide valuable insights into the overall impact of complete CA-V deficiency in organisms.^13^ However KO models fall short in addressing the specific timing, mechanisms, and causality of CA-V dysregulation, particularly in relation to mitochondrial dysfunction. A second approach involves inhibiting CA-V within isolated mitochondria.^8,19–21^ While this method offers kinetic and biochemical data for CA-V, it requires that mitochondria are extracted from cells, leaving the interactions of CA-V within the broader cellular context unexplored. Finally, CA-V-specific inhibitors have been pursued and tested extensively *in vitro*, some with CA-V selectivity ratios as high as 3800, though their activation cannot be controlled in a spatiotemporal manner.^22–34^

In this study, we report a novel photocaged CA inhibitor (PhotoCaged Ethoxzolamide Mitochondria-targeted, PCEM) that can be selectively delivered to mitochondria without inhibiting cytosolic or other CA isoforms in the process (Fig. 1). This tool uses an aryl sulfonamide (–SO_2_NH_2_) for coordination to the Zn^2+^ in the CA active site. This ligand is blocked from interacting with CA by a photoremovable protecting group (coumarin) at one end and linked to a mitochondrial-directing group (pyridinium) at the other end. Aryl sulfonamides are well-established and highly selective inhibitor motifs known to coordinate to the Zn^2+^ ion of CA, thereby obstructing substrate access and inhibiting enzyme activity.^20,35^ The photoprotecting group coumarin exhibits a high quantum yield and a visible light activation wavelength, enhancing its biocompatibility, and can be visualized by fluorescence microscopy.^36–38^ The pyridinium moiety is a delocalized lipophilic cation that targets mitochondria due to its ability to penetrate membranes and its attraction to the negative potential of the mitochondrial matrix.^39,40^ In our approach, the mitochondrial-directing group will shuttle the molecule into the mitochondria as the protecting group simultaneously prevents it from binding to extracellular and cytoplasmic CAs. Once the molecule is within the mitochondria, photoirradiation is used to remove the protecting group, thus exposing the sulfonamide and allowing it to interact with and inhibit mitochondrial CA-V isoforms. To date, this is the first reported photoactivable inhibitor for targeting mitochondrial CA-V isoforms.^41^ By providing spatial and temporal control over inhibitor activation, this design enables the selective inhibition of a specific CA isoform within its natural cellular environment.

**Fig. 1:**
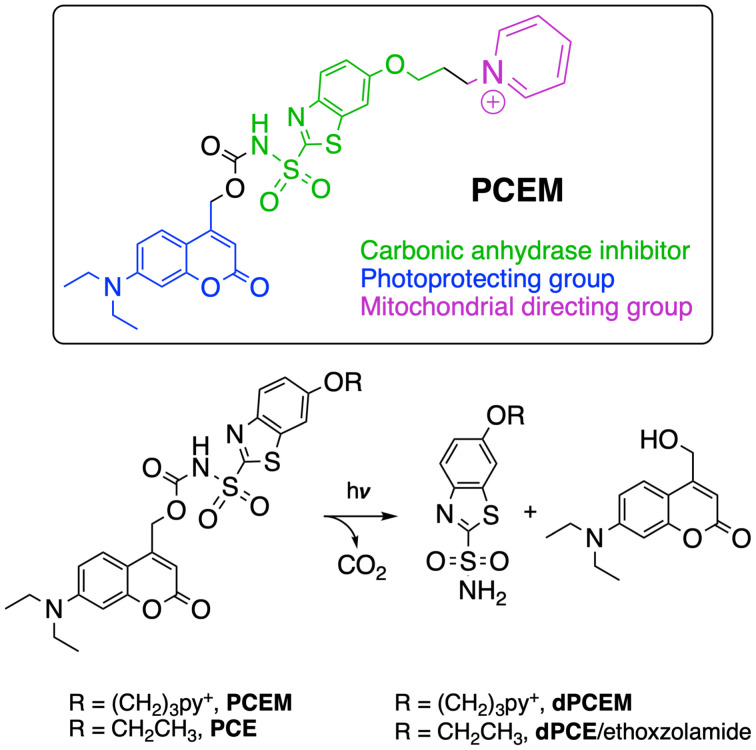
Design and structure of PCEM, a photocaged probe for targeting mitochondrial carbonic anhydrase V isoforms and light-mediated deprotection of PCEM and control molecule PCE. Note that the structure of dPCE is the same as known CA inhibitor ethoxzolamide.

To optimize our photocaged CA-V inhibitor, we built a homology model for human CA-VA using murine CA-VA (PDB 1URT)^42^ due to their 79% sequence similarity (Fig. S1†). While surface residues showed divergence, active site residues were highly conserved. The active site contains a number of flexible loops; however, the model generated was suitable for docking studies to refine probe design. Using our hCA-VA model, we conducted docking studies to evaluate probe interactions. We first tested triphenyl phosphonium (R-PPh_3_^+^), a common mitochondrial-targeting group,^43^ but observed no significant binding regardless of linker length, likely due to steric clashes at the active site entrance. To address this, we replaced R-PPh_3_^+^ with pyridinium (R-Py^+^), which is less bulky and hydrophobic in nature. A three-carbon linker was identified as optimal, balancing Zn^2+^ binding with solubility in aqueous solutions. The docked structure of this final probe design (Fig. 2) shows a coordination bond between the active site Zn^2+^ and the deprotonated sulfonamide moiety, as well as hydrogen bonding interactions with Thr235 and Gln128 in the active site cavity.

**Fig. 2:**
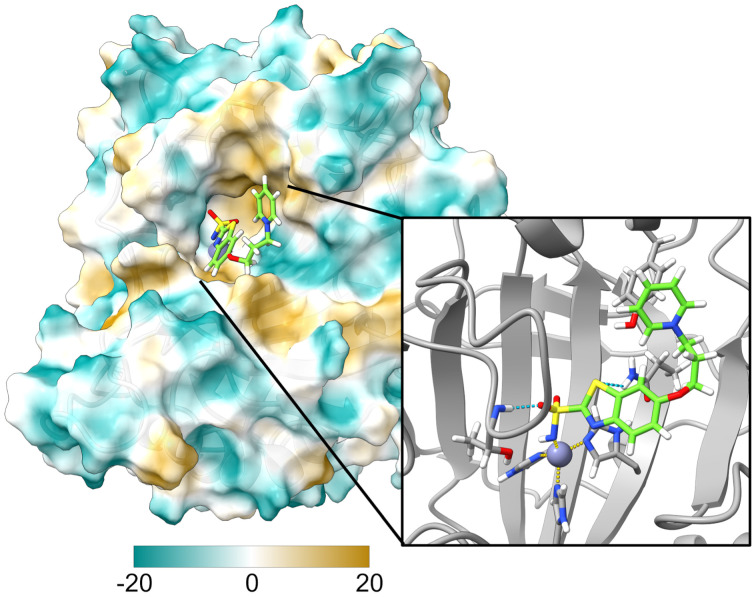
Docked structure of final PCEM probe in hCA-VA homology model. Inset: enlarged view to highlight active site metal coordination (dashed yellow) and hydrogen bonding (dashed blue) interactions.

In addition to our final photocaged inhibitor PCEM, we also synthesized a control molecule, PCE, lacking the mitochondrial-targeting group. The full syntheses of both PCEM and PCE are detailed in Scheme S1† and the deprotection reaction is shown in Fig. 1. The final PCE and PCEM structures were obtained following coupling of ethoxzolamide or the pyridinium-containing derivative 9 with coumarin 3 in the presence of 4-nitrophenyl chloroformate and DMAP. Irradiation with 360–400 nm light should result in CO_2_ release and regeneration of coumarin 3 and the corresponding sulfonamides dPCE and dPCEM. We note that the structures of dPCE and ethoxzolamide are identical, however we will refer to the photodeprotection product of PCE as dPCE in characterization studies to distinguish samples treated with PCE and subsequently deprotected from those directly treated with ethoxzolamide.

The photophysical properties of PCEM and PCE were assessed in aqueous buffer solution using absorbance and fluorescence spectroscopies and compounds were photoactivated using a Xenon lamp equipped with 365 nm or 410 nm bandpass filters (Fig. S2–S6†). In the absorbance spectrum of PCE, a pronounced absorption band at 400 nm was observed, which decreased upon irradiation with either 365 nm or 410 nm light. A similar trend was observed in the fluorescence emission, with the peak at *λ*_em_ = 500 nm (*λ*_ex_ = 410 nm) diminishing slightly following photoirradiation with 365 nm and 410 nm light. We opted to proceed with 410 nm light for further investigations due to higher biocompatibility and slightly faster deprotection kinetics (Fig. S6†). Photodeprotection of PCE and PCEM and characterization of their resultant products were acheived through LC/MS analysis (Fig. S7–S10†). Conversion of both molecules to coumarin and dPCE or dPCEM was observed without the formation of undesired byproducts.

We used a NPA (4-nitrophenyl acetate) assay^44^ to assess the inhibitory properties of our probes (PCEM and PCE) and their photo-deprotected analogs (dPCEM and dPCE) after irradiation with 410 nm light. Fig. S11† depicts the increase of absorbance at 400 nm over time when bCA-II was subjected to 1 equivalent of PCE and PCEM in the absence of light. This change is similar to the control where protein was not treated with any inhibitor, showing the designed probes exhibit no inhibitory properties towards bCA-II. When the probes are irradiated with 410 nm light, to generate dPCE and dPCEM before incubation with bCA-II and NPA, the change in absorbance with time decreases substantially. This trend is similar to the case when bCA-II was treated with ethoxzolamide, proving the inhibitory properties of the probes can be released upon photoirradiation. For quantitative comparisons between the protected and deprotected forms of the probe, IC_50_ values were assessed (Fig. 3) and compared to ethoxzolamide (IC_50_ = 0.1 μM). Prior to deprotections, we observed an IC_50_ of 13.6 μM for PCE and much higher for PCEM (data could not be fitted to an IC_50_ value). After deprotection, the IC_50_ values decreased to 1.1 μM for dPCE and 2.9 μM for dPCEM, respectively, suggesting that both dPCE and dPCEM are effective inhibitors for CA, and the presence of the mitochondrial-targeting R-Py^+^ moiety in dPCEM does not prevent CA inhibition.

**Fig. 3:**
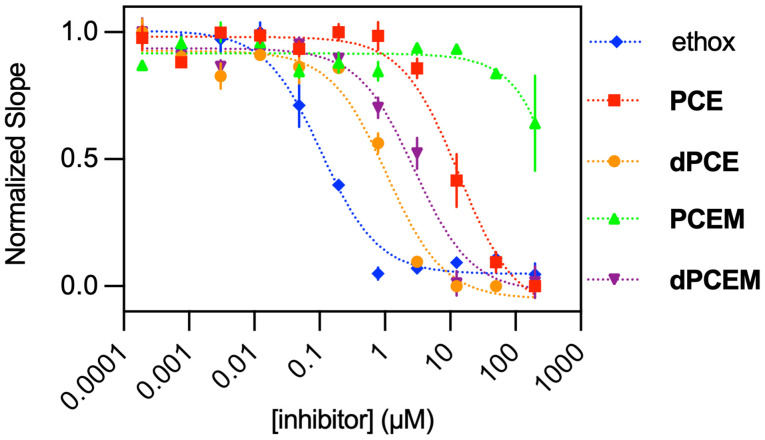
IC_50_ data for inhibition of NPA hydrolysis by bCA-II in the presence of PCEM, PCE, dPCEM, and dPCE. Data for PCEM could not be fitted to an IC_50_ value.

After demonstrating the distinct interaction patterns of PCE and PCEM with isolated bCA-II protein in both protected and deprotected states, we proceeded to investigate the ability of PCEM to target CA-V in isolated mitochondria and in whole cells. Our goals were to demonstrate that our probes are capable of inhibiting the mitochondrial isoforms of CA and show that our combined targeting and photocaging strategy promotes localization of our probes to mitochondria and prevents interaction of our probes with non-mitochondrial CAs.

CAs are essential enzymes that catalyze the conversion of carbon dioxide and water into bicarbonate and protons, playing pivotal roles in pH regulation and CO_2_ transport. These processes influence metabolic pathways, including citrulline production in mitochondria. Given that much more is understood about the biology of CA-VA, we focused our studies on this mitochondrial isoform.^4^ It has been reported that inhibition of mitochondrial CA-VA leads to a significant reduction in citrulline levels, likely due to disruption of bicarbonate balance, which affects both the urea cycle and arginine metabolism.^45^ Here, we quantified the total citrulline content in cells using a colorimetric assay in which an indicator reacts with citrulline to form a chromophore and absorbance is measured at 550 nm.^46^ As HepG2 cells express high levels of CA-VA in their mitochondria,^12^ testing the ability of our probes to inhibit citrulline production in isolated mitochondria provides a means of assessing the ability of PCEM and PCE to inhibit this CA isoform. We expect both probes to reduce citrulline production if they are able to effectively bind to the CA-VA active site. We performed a citrulline assay in isolated mitochondria from HepG2 cells following treatment of cells with 50 μM PCE, PCEM, or DMSO (control), alongside 50 μM ornithine to stimulate citrulline synthesis. At these concentrations, protected and deprotected probes do not show cytotoxicity over a 24 h time period (Fig. S12†). Following treatment with either PCE or PCEM, cells were irradiated with 410 nm light and then incubated overnight. As shown in Fig. 4A, samples exposed to dPCE and dPCEM exhibited significantly lower citrulline levels compared to the DMSO control, confirming effective CA inhibition. As expected, no significant difference in citrulline production was observed between dPCE and dPCEM treatments, which indicates both probes can inhibit CA-VA.

**Fig. 4:**
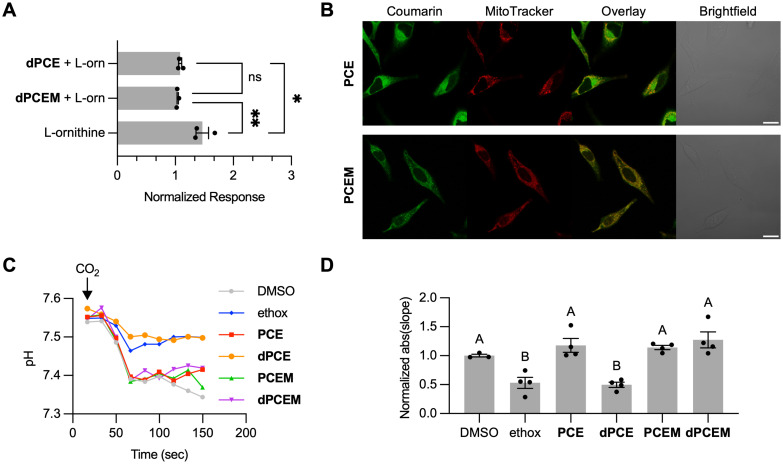
Cellular studies with PCEM and PCE. (A) Inhibition of citrulline production by dPCE and dPCEM in presence of l-ornithine (B) Confocal images showing localization of PCE and PCEM (green, 1 μM, *λ*_ex/em_: 405/450–530 nm) and MitoTracker Red (red, 0.1 μM, *λ*_ex/em_: 560/580–700 nm) in HeLa cells. Scale bar = 20 μm (C) Change in cytosolic pH over time following addition of CO_2_ for cells incubated with DMSO, ethoxzolamide (ethox), (d)PCE or (d)PCEM. (D) Rate of cytosolic pH change following addition of CO_2_. The average absolute value of the slope for pH between 33 and 66 s in each condition is shown (*n* = 4). A one-way ANOVA was performed to assess the difference in average slope between all treatments. Bars labeled ‘A’ and ‘B’ show groups that are statistically different (*p* < 0.05). All studies were performed in FluoroBrite DMEM at room temperature.

We then studied if addition of the mitochondrial targeting Py^+^ group is effective in directing the probe to mitochondria in whole live cells. For these studies, we focused on HeLa cells, as their flattened morphology in cell culture is more amenable to colocalization studies compared to HepG2 cells, which tend to form clumps of rounded cells.^47^ To confirm localization of PCEM, we conducted confocal imaging following the treatment of HeLa cells with MitoTracker Red^48^ and either PCE or PCEM. As shown in Fig. 4B, PCE shows a generalized cytosolic localization, with poor co-localization with MitoTracker (Pearson's *R* = 0.2, Fig. S13†). Conversely, cells incubated with PCEM display more localized staining and higher co-localization with MitoTracker (Pearson's *R* = 0.4). This correlation difference was statistically significant and supports improved mitochondrial localization of PCEM compared to PCE.

To demonstrate the differential inhibition properties of PCEM and PCE in whole cells, we examined how treatment with these probes affects the ability of cytosolic CAs (CA-II) to respond to exogenous CO_2_ perturbation. Following CO_2_ exposure, extracellular CO_2_ is expected to diffuse through the cell membrane, raising its cytoplasmic concentration, where it is converted to carbonic acid by cytoplasmic CAs, resulting in a decrease in cytosolic pH.^49^ Inhibition of cytoplasmic CA reduces the rate of intracellular pH change resulting from the introduction of CO_2_.^50–52^ pH changes were tracked over time in the presence of our probes to evaluate real-time inhibition activity. If our mitochondrial targeting strategy is effective, treatment with PCEM should not affect the cytosolic pH response.

HeLa cells were treated with pHrodo dye and incubated with PCE, dPCE, PCEM, dPCEM, DMSO, and ethoxzolamide. CO_2_-saturated water was then introduced into the cell suspension, and fluorescence intensity was monitored over time using flow cytometry, with the changes in pHrodo fluorescence representing variations in intracellular pH. As illustrated in Fig. 4C, CO_2_ introduction in the presence of PCE and PCEM led to a decrease in intracellular pH, similar to the DMSO control. However, after deprotection, dPCEM showed no significant change compared to protected PCEM, whereas for dPCE pH decreased more gradually, similar to ethoxzolamide. This is quantified in Fig. 4D, in which the slopes of the pH decrease are compared. This confirms that introduction of the Py^+^ group effectively localizes PCEM and dPCEM to the mitochondria, and thus does not inhibit cytosolic pH change, whereas the untargeted dPCE has a broad CA inhibitory effect, including inhibition of CAs in the cytosol.

## Conclusions

We have demonstrated that by combining photocaging and intracellular targeting strategies, a carbonic anhydrase active site inhibitor can be effectively targeted to an intracellular compartment and does not globally inhibit the activity of carbonic anhydrases in cells. Additionally, optimizing probe design to incorporate a sterically compact targeting group allows the photocaged inhibitor to still interact with the protein after activation with light. Next generation designs could incorporate the targeting moiety onto the photoprotecting group, which could improve interactions between the target protein and the deprotected inhibitor. Due to the ease of modifying metal-binding inhibitors with photocaging groups and the abundance of subcellular targeting strategies, we anticipate that this approach will be useful to target other metalloenzymes within many subcellular spaces including mitochondria, the endoplasmic reticulum, the nucleus, and the extracellular membrane.

## Author contributions

N. M.: conceptualization, investigation, formal analysis, methodology, validation, visualization, writing – original draft. K. A.: conceptualization, investigation, formal analysis, methodology, validation, visualization, writing – original draft. S. P.: investigation, formal analysis, methodology, validation, visualization, writing – original draft. D. H.: investigation, formal analysis, validation, visualization, writing – review and editing. E. Y.: investigation, methodology, writing – review and editing. D. K.: investigation, methodology, writing – review and editing. E. Q.: conceptualization, formal analysis, visualization, writing – original draft, project administration, resources, funding acquisition, supervision.

## Conflicts of interest

There are no conflicts to declare.

## Supplementary Material

DT-054-D5DT01161B-s001

## Data Availability

Data for this article are available at the Texas Data Repository at https://dataverse.tdl.org/.
