# Supplementary material for: A subcellularly targeted photocaged inhibitor for mitochondrial carbonic anhydrase V
Source: Dalton Trans. 2025 Jul 4;54(28):10862–7. doi: 10.1039/d5dt01161b (PMC12226970; doi:10.1039/d5dt01161b)
Supplement: DT-054-D5DT01161B-s001 [file DT-054-D5DT01161B-s001.pdf]

Supplementary Information (SI) for Dalton Transactions.
This journal is © The Royal Society of Chemistry 2025

Supporting information for

A Subcellularly Targeted Photocaged Inhibitor for Mitochondrial Carbonic Anhydrase V

Noushaba Mafy#, Kanchan Aggarwal#, Sky Price, Dorothea B. Hudson, Elva Ye, Divya Kolli, Emily L. Que*

Department of Chemistry, The University of Texas at Austin

Table of Contents

	Page
General experimental Information	S2
Synthesis	S3-S7
Additional Experimental Descriptions	S7-S10
**Figure S1** Quality assessment of Model 3 homology structure of hCA-VA	S11
**Figure S2** UV-vis monitoring of photodeprotection of **PCE**	S12
**Figure S3** UV-vis monitoring of photodeprotection of **PCEM**	S13
**Figure S4** Fluorescence monitoring of photodeprotection of **PCE**	S14
**Figure S5** Fluorescence monitoring of photodeprotection of **PCEM**	S15
**Figure S6** Photodeprotection kinetics of **PCE** using 365 nm and 410 nm light	S16
**Figure S7** LC/MS analysis of **PCE** prior to photodeprotection	S17
**Figure S8** LC/MS analysis of **PCEM** prior to photodeprotection	S18
**Figure S9** LC/MS analysis of **PCE** following partial photodeprotection	S19
**Figure S10** LC/MS analysis of **PCEM** following partial photodeprotection	S20
**Figure S11** NPA assay time course data	S21
**Figure S12** MTT assays	S22
**Figure S13** Cellular colocalization analysis	S23
References	S23
HRMS data and NMR spectra (^{1}H and ^{13}C) for **1–9**, **PCE**, **PCEM**	S24-S45

1. General experimental information

All solvents and chemicals were purchased from Sigma-Aldrich and Fisher Scientific and used as received for synthesis, characterization, and analysis. Bovine carbonic anhydrase was obtained from Sigma-Aldrich. Spectroscopic and protein studies were conducted using a 0.05 M HEPES buffer containing 0.1 M KNO_3 (pH 7.2). Probe stock solutions were prepared in DMSO, and all studies were performed in the dark to prevent light exposure.

^1H NMR and ^{13}C NMR spectra were recorded in deuterated solvents obtained from Cambridge Isotope Laboratories (Cambridge, MA) using an Agilent MR 400 NMR spectrometer operating at 400 MHz. Chemical shifts were calibrated relative to the solvent peak and reported in ppm. Absorbance spectroscopic studies were carried out on an Agilent Cary 60 UV-Vis spectrophotometer. Fluorescence spectroscopic measurements were performed using an Agilent Cary Eclipse fluorescence spectrofluorometer. Sample irradiation was achieved using an Asahi Xe lamp equipped with bandpass filters of 365 nm (FWHM 12 nm), 460 nm (FWHM 11 nm) from Asahi Spectra, and 410 nm (bandwidth 10 nm) from Thorlabs. Absorbance measurements for IC_{50} experiments were performed on a Molecular Devices SpectraMax iD5 microplate reader.

Walk-up LC-MS and high-resolution electrospray ionization (ESI) mass spectrometric analyses were conducted by the Mass Spectrometry Facility at the Department of Chemistry, UT Austin.

HepG2 and HeLa cell lines were purchased from ATCC and cultured as recommended. Mitochondria were isolated using the Mitochondria Isolation Kit from Miltenyi Biotec, designed to obtain functional and viable mitochondria from human cells or tissues. The Homocitrulline/Citrulline Assay Kit from Cell Biolabs Inc. was used for citrulline assay.

2. Synthesis:

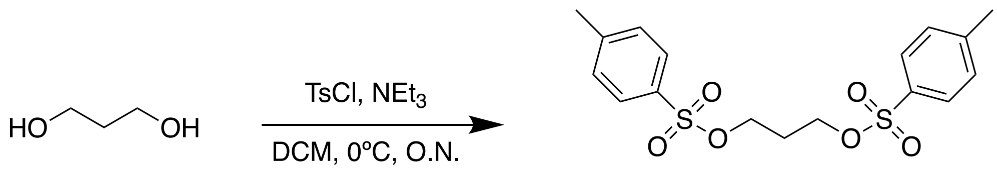

1

Synthesis of Compound **1**: To propanediol (0.5 g, 6.57 mmol) in 25 mL dichloromethane was added triethylamine (3.6 mL, 26 mmol) and the solution was cooled to 0 ºC. Tosyl chloride (2.52 g, 13 mmol) was dissolved in 25 mL DCM and the solution was added dropwise over the course of one hour. The reaction was allowed to warm to room temperature and stirred overnight. The reaction mixture was washed with DI H_2O (3 x 15 mL) and brine (15 mL) and the organic layer was dried over magnesium sulfate, then concentrated under reduced pressure. The crude product was purified on SiO_2 by flash chromatography (30% EtOAc/Hex) to yield the product as a white solid (1.78 g, 71%): ^{1}H NMR (500 MHz, $CDCl_3$) δ 7.75 (d, *J* = 8.2 Hz, 4H), 7.35 (d, *J* = 7.9 Hz, 4H), 4.06 (t, *J* = 5.9 Hz, 4H), 2.46 (s, 6H), 2.00 (p, *J* = 5.9 Hz, 2H) ppm; ^{13}C NMR (100 MHz, $CDCl_3$) δ 145.2, 132.8, 130.1, 128.0, 66.0, 28.8, 21.8 ppm; HRMS ESI$^+$ *m/z* calc'd for [$C_{17}H_{20}O_6S_2$ + Na^+] (407.0590, observed; 407.0594, expected).

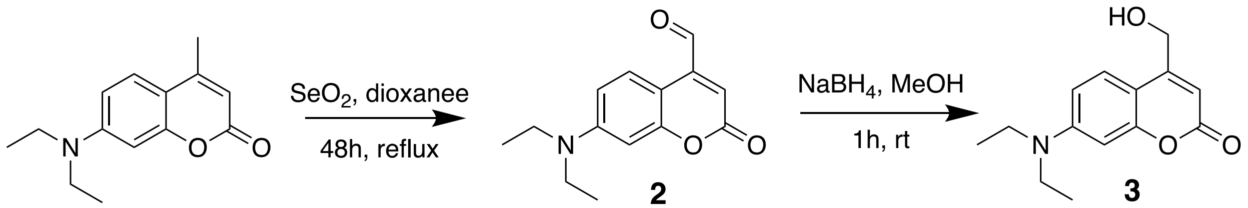

2 **3**

Synthesis of Compound **2**: Selenium dioxide (0.72 g, 6.50 mmol) was added to a solution of 7-(diethylamino)-4-methylcoumarin (1.0 g, 4.33 mmol) in dioxane (25 mL). The reaction mixture was refluxed for 48 hours, then cooled to room temperature, filtered through celite, and concentrated. The crude reaction mixture was purified on SiO_2 by flash chromatography (DCM) to yield the product as a red solid (0.335 g, 32%): ^{1}H NMR (400 MHz, $CDCl_3$) δ 10.03 (s, 1H), 8.31 (d, *J* = 9.2 Hz, 1H), 6.64 (dd, *J* = 9.2, 2.6 Hz, 1H), 6.53 (d, *J* = 2.6 Hz, 1H), 6.46 (s, 1H), 3.43 (q, *J* = 7.2 Hz, 5H), 1.22 (t, *J* = 7.1 Hz, 8H) ppm; ^{13}C NMR (100 MHz, $CDCl_3$) δ 192.6, 162.0, 157.5, 151.1, 144.0, 127.2, 117.6, 109.7, 103.9, 97.9, 45.0, 12.6 ppm; HRMS ESI$^+$ *m/z* calc'd for [$C_{14}H_{15}NO_3$ + Na^+] (268.0948, observed; 268.0944, expected).

Synthesis of Compound **3**: Compound **2** (200 mg, 0.82 mmol) was dissolved in 5 mL dry ethanol, then sodium borohydride (33 mg, 0.87 mmol) was added. The reaction was stirred in the dark at room temperature for 1.5 hours, at which point the reaction was quenched with 1M HCl (2 mL), diluted with DI H_2O (3 mL), and then extracted into DCM (3 x 10 mL). The organic extracts were dried over $MgSO_4$ and concentrated to yield the product as a yellow solid (200 mg, 99%): ^{1}H NMR (500 MHz, $CDCl_3$) δ 7.30 (d, *J* = 8.8 Hz, 1H), 6.50 (d, *J* = 54.7 Hz, 2H), 6.29 (s, 1H), 4.81 (s, 2H), 3.36 (q, *J* = 7.1 Hz, 4H), 1.16 (t, *J* = 7.0 Hz, 6H) ppm; ^{13}C NMR (100 MHz, $CDCl_3$) δ 163.1, 156.0, 155.7, 124.5, 60.7, 44.9, 29.8, 12.5 ppm; HRMS ESI$^+$ *m/z* calc'd for [$C_{14}H_{17}NO_3$ + Na^+] (270.1099, observed; 270.1101, expected).

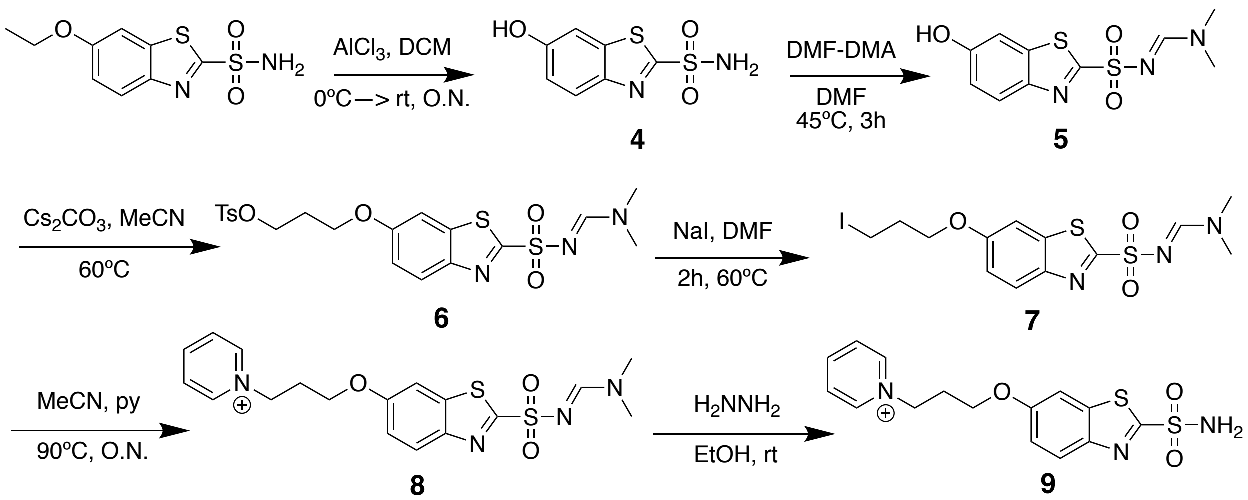

Synthesis of Compound **4**: Ethoxzolamide (400 mg, 1.55 mmol) was dissolved in dry DCM (8 mL) and the mixture was cooled to 0 ºC before aluminum chloride (737 mg, 5.53 mmol) was added portion-wise over about 15 minutes. The reaction was warmed to room temperature and stirred overnight, then quenched with 3M HCl (20 mL). The precipitate was filtered and washed with DI H_2O, then purified on SiO_2 by flash chromatography (40-60% EtOAc/Hex) to yield the product as a pale yellow solid (348 mg, 97%): ^{1}H NMR (400 MHz, MeOD) δ 7.87 (d, *J* = 8.9 Hz, 1H), 7.32 (d, *J* = 2.3 Hz, 1H), 7.06 (dd, *J* = 8.9, 2.4 Hz, 1H) ppm; ^{13}C NMR (100 MHz, MeOD) δ 166.5, 158.8, 147.0, 139.4, 126.1, 118.6, 107.3 ppm; HRMS ESI$^+$ *m*/*z* calc'd for [$C_7H_6N_2O_3S_2$ + Na^+] (252.9710, observed; 252.9712, expected).

Synthesis of Compound **5**: Compound **4** (55 mg, 0.24 mmol) was dissolved in DMF (0.4 mL), then dimethylformamide dimethyl acetal (58 µL, 0.43 mmol) was added. The reaction was heated to 45 ºC for 3 hours, then concentrated under reduced pressure. The crude reaction mixture was purified on SiO_2 by flash chromatography (5% MeOH/DCM) to yield the product as a yellow solid (67.5 mg, 99%): ^{1}H NMR (400 MHz, DMSO-*d*$_6$) δ 10.19 (s, 1H), 8.33 (s, 1H), 7.90 (d, *J* = 8.9 Hz, 1H), 7.44 (d, *J* = 2.4 Hz, 1H), 7.05 (dd, *J* = 8.9, 2.5 Hz, 1H), 3.19 (s, 2H), 2.95 (s, 2H) ppm; ^{13}C NMR (100 MHz, DMSO-*d*$_6$) δ 164.6, 160.9, 157.2, 145.5, 137.6, 125.1, 117.6, 106.8, 41.3, 40.4, 35.5 ppm; HRMS ESI$^+$ *m*/*z* calc'd for [$C_{10}H_{11}N_3O_3S_2$ + Na^+] (308.0135, observed; 308.0134, expected).

Synthesis of Compound **6**: To cesium chloride (100 mg, 0.31 mmol) was added a solution of compound **5** in dry MeCN (5 mL, 38.5 mM). The mixture was allowed to react for about ten minutes, at which point compound 1 (80 mg, 0.21 mmol) was added and the reaction was heated to 60 ºC. After 3 hours, the reaction was quenched with sat. aq. NH_4Cl (5 mL) and extracted into DCM (3 x 25 mL). The organic extracts were dried over Na_2SO_4 and concentrated before the crude material was purified on SiO_2 by flash chromatography (1% MeOH/DCM) to yield the product as a pale yellow solid (42 mg, 41%): ^{1}H NMR (400 MHz, $CDCl_3$) δ 8.31 (s, 1H), 7.95 (dd, *J* = 9.0, 2.6 Hz, 1H), 7.73 (d, *J* = 8.1 Hz, 2H), 7.25 – 7.16 (m, 3H), 6.99 (dt, *J* = 9.0, 2.5 Hz, 1H), 4.25 (t, *J* = 5.8 Hz, 2H), 4.02 (t, *J* = 5.7 Hz, 2H), 3.21 (s, 3H), 3.08 (s, 3H), 2.31 (s, 3H), 2.16 (p, *J* = 5.4 Hz, 2H) ppm; ^{13}C NMR (100 MHz, $CDCl_3$) δ 165.4, 161.1, 158.0, 147.2, 145.0, 138.3, 132.8,

129.9, 127.9, 125.6, 117.5, 104.4, 66.9, 63.9, 42.1, 36.1, 28.8, 21.7 ppm; HRMS ESI$^+$ *m*/*z* calc'd for [$C_{20}H_{23}N_3O_6S_3$ + Na^+] (520.0638, observed; 520.0641, expected).

Synthesis of Compound **7**: To compound **6** (109 mg, 0.22 mmol) in DMF (5 mL) was added NaI (164 mg, 1.1 mmol). The reaction was heated to 60 ºC and stirred for 5 hours, at which point the DMF was evaporated under reduced pressure. The crude reaction mixture was redissolved in EtOAc (50 mL) and washed copiously with brine. The organic extract was dried over Na_2SO_4 and concentrated to yield the product as a yellow solid (90 mg, 91%): ^{1}H NMR (400 MHz, $CDCl_3$) δ 8.32 (s, 1H), 8.01 (d, *J* = 9.0 Hz, 1H), 7.36 (d, *J* = 2.5 Hz, 1H), 7.15 (dd, *J* = 9.1, 2.5 Hz, 1H), 4.13 (t, *J* = 5.8 Hz, 2H), 3.39 (t, *J* = 6.6 Hz, 2H), 3.22 (s, 3H), 3.10 (s, 3H), 2.32 (p, *J* = 6.2 Hz, 2H) ppm; ^{13}C NMR (100 MHz, $CDCl_3$) δ 165.3, 161.1, 158.3, 147.4, 138.5, 125.8, 117.7, 104.6, 68.2, 42.1, 36.1, 32.8, 2.3 ppm; HRMS ESI$^+$ *m*/*z* calc'd for [$C_{13}H_{16}IN_3O_3S_2$ + Na^+] (475.9567, observed; 475.9570, expected).

Synthesis of Compound **8**: To compound **7** (93 mg, 0.21 mmol) in dry MeCN (3 mL) was added pyridine (33 µL, 0.42 mmol). The reaction was heated to 90 ºC and left to react for 24 hours, then concentrated. The crude reaction mixture was purified on C18 SiO_2 by reverse-phase flash chromatography (25% MeCN/H_2O with 0.1% formic acid) to yield the product as an orange solid (58.5 mg, 70%): ^{1}H NMR (400 MHz, DMSO-*d*$_6$ with 10% D_2O) δ 9.22 (d, *J* = 5.8 Hz, 2H), 8.64 (t, *J* = 7.7 Hz, 1H), 8.46 (s, 1H), 8.38 (s, 1H), 8.18 (t, *J* = 6.6 Hz, 2H), 8.01 (d, *J* = 9.0 Hz, 1H), 7.72 (d, *J* = 2.6 Hz, 1H), 7.01 (dd, *J* = 9.1, 2.3 Hz, 1H), 4.87 (t, *J* = 6.6 Hz, 2H), 4.22 (t, *J* = 5.5 Hz, 2H), 3.25 (s, 3H), 3.00 (s, 3H), 2.55 (p, 2H) ppm; ^{13}C NMR (100 MHz, DMSO-*d*$_6$ with 10% D_2O) δ 166.0, 160.9, 157.3, 146.5, 145.6, 145.2, 137.5, 128.0, 125.0, 117.5, 105.5, 65.7, 58.8, 41.3, 35.5, 29.8 ppm; HRMS ESI$^+$ *m*/*z* calc'd for [$C_{18}H_{21}N_4O_3S_2^+$] (405.1058, observed; 405.1050, expected). Note the presence of an exchangeable proton at 8.46 ppm, and the partial obscurement of the pentet at 2.55 ppm by the solvent residual signal.

Synthesis of Compound **9**: To compound **8** (36.8 mg, 0.09 mmol) in EtOH (2 mL) was added 60% aqueous hydrazine monohydrate (45 µL, 0.54 mmol). The reaction was stirred in a capped vial for 20 minutes before being concentrated under reduced pressure. The crude reaction mixture was purified on C18 SiO_2 by reverse-phase flash chromatography (20% MeOH/H_2O with 0.1% formic acid) to yield the product as a pale yellow solid (14.6 mg, 46%): ^{1}H NMR (400 MHz, DMSO-*d*$_6$ with 10% D_2O) δ 8.98 (d, *J* = 5.9 Hz, 2H), 8.54 (t, *J* = 7.8 Hz, 1H), 8.36 (s, 1H), 8.05 (t, *J* = 7.0 Hz, 2H), 7.98 (d, *J* = 9.0 Hz, 1H), 7.60 (d, *J* = 2.5 Hz, 1H), 6.99 (dd, *J* = 9.0, 2.5 Hz, 1H), 4.77 (t, *J* = 6.8 Hz, 2H), 4.16 (t, *J* = 5.6 Hz, 2H), 2.45 (p, *J* = 6.3 Hz, 2H) ppm; ^{13}C NMR (100 MHz, DMSO-*d*$_6$ with 10% D_2O) δ 166.9, 158.1, 146.9, 146.5, 145.4, 138.2, 128.8, 125.8, 118.5, 106.0, 66.3, 59.9, 30.4 ppm; HRMS ESI$^+$ *m*/*z* calc'd for [$C_{15}H_{16}N_3O_3S_2^+$] (350.0632, observed; 350.0628, expected). Note the presence of an exchangeable proton at 8.36 ppm, and the partial obscurement of the pentet at 2.45 ppm by the solvent residual signal.

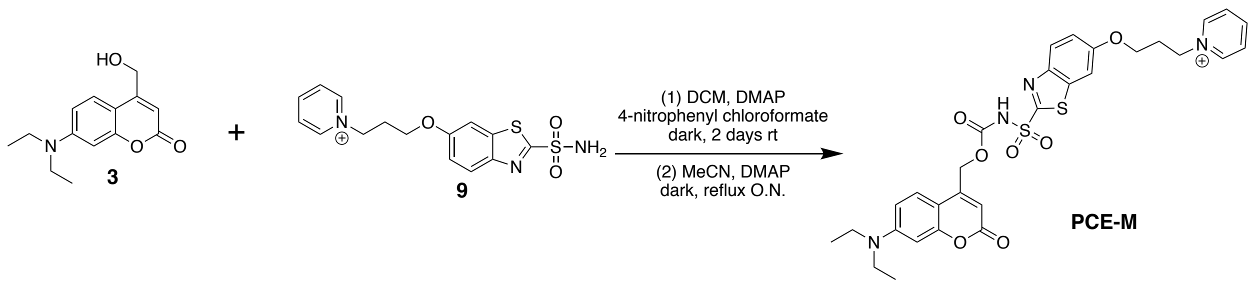

Synthesis of **PCE-M:** Compound **3** (300 mg, 1.21 mmol) was added in dry DCM (24 mL) along with DMAP (318 mg, 2.60 mmol) and 4-nitrophenyl chloroformate (558 mg, 2.77 mmol) in the dark. The solution mixture was stirred for 2 days until the reactant was reacted completely, as monitored by TLC. The reaction mixture was concentrated under vacuum and suspended in acetonitrile. Compound **9** (424 mg, 1.21 mmol) and DMAP (300 mg, 2.46 mmol) was added to the mixture and refluxed overnight. The solvent was then evaporated and product was separated through reverse phase column chromatography to obtain the product (755 mg, quant.). ^{1}H NMR (400 MHz, DMSO-d_6) δ 9.13 (dd, J = 5.6, 1.5 Hz, 2H), 8.61 (t, J = 7.8 Hz, 1H), 8.15 (dd, J = 7.8, 6.5 Hz, 2H), 7.90 (d, J = 9.0 Hz, 1H), 7.56 (d, J = 2.6 Hz, 1H), 7.38 (d, J = 9.0 Hz, 1H), 6.89 (dd, J = 9.0, 2.6 Hz, 1H), 6.63 (dd, J = 9.0, 2.6 Hz, 1H), 6.50 (d, J = 2.6 Hz, 1H), 5.92 (s, 1H), 5.00 (s, 2H), 4.82 (t, J = 6.7 Hz, 2H), 4.17 (t, J = 5.6 Hz, 2H), 2.67 (p, J = 1.8 Hz, 1H), 2.33 (p, J = 1.9 Hz, 1H), 1.10 (t, J = 7.0 Hz, 6H) ppm; ^{13}C NMR (126 MHz, DMSO-d_6) δ 170.3, 160.8, 157.5, 156.5, 155.7, 152.8, 150.3, 146.6, 145.7, 145.1, 137.5, 128.0, 125.3, 124.4, 116.3, 108.7, 105.5, 105.1, 104.4, 96.8, 65.5, 61.0, 59.0, 44.0, 29.8, 12.3 ppm; HRMS ESI^{+} *m/z* calc'd for [$C_{30}H_{31}N_4O_7S_2^{+}$] (623.1628, observed; 623.1629, expected). Note that a quartet corresponding to 4H is obscured by the solvent residual signal at 3.33 ppm.

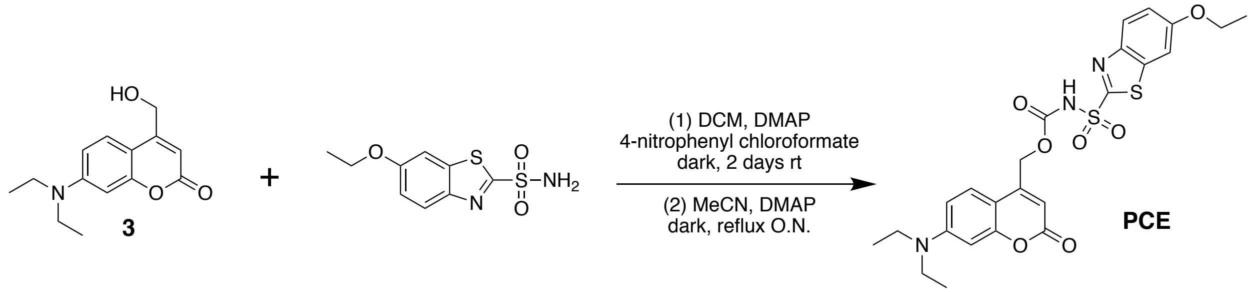

Synthesis of **PCE:** Compound **3** (100 mg, 0.40 mmol) was added in dry DCM along with DMAP (106 mg, 0.87 mmol) and 4-nitrophenyl chloroformate (185 mg, 0.92 mmol) in the dark. The solution mixture was stirred for 2 days until the reactant was reacted completely, as monitored by TLC. The reaction mixture was concentrated under vacuum and suspended in acetonitrile. Ethoxazolamide (150 mg, 0.58 mmol) and DMAP (100 mg, 0.82 mmol) was added to the mixture and refluxed overnight. The solvent was then evaporated and product was separated through reverse phase column chromatography to obtain the product (211 mg, quant.). ^{1}H NMR (400 MHz, DMSO-d_6) δ 7.90 (d, J = 9.0 Hz, 1H), 7.62 (d, J = 2.6 Hz, 1H), 7.35 (d, J = 9.1 Hz, 1H), 7.10 (dd, J = 9.0, 2.6 Hz, 1H), 6.60 (dd, J = 9.0, 2.6 Hz, 1H), 6.48 (d, J = 2.5 Hz, 1H), 5.92 (d, J = 1.4 Hz, 1H), 4.98 (d, J = 1.4 Hz, 2H), 4.08 (q, J = 6.9 Hz, 2H), 3.39 (q, J = 7.0 Hz, 4H), 1.34 (t, J = 7.0 Hz, 3H), 1.08 (t, J = 7.0 Hz, 6H) ppm; ^{13}C NMR (126 MHz, DMSO-d_6) δ 169.9, 160.9, 157.6, 157.2, 155.7, 152.8, 150.3, 146.3, 137.6, 125.3, 124.4, 116.6, 108.7, 105.5, 105.0,

104.6, 96.8, 63.8, 61.1, 44.0, 14.6, 12.3 ppm; HRMS ESI$^+$ *m/z* calc'd for [$C_{24}H_{26}N_3O_7S_2^+$] (532.1207, observed; 532.1207, expected).

2. Photospectral properties

The photocleaving process of molecules (10mM stock in DMSO) were investigated by electronic absorption spectroscopy. The absorbance and fluorescence spectrum were taken in methanol and 0.05 M HEPES buffer containing 0.1 M KNO_3 (pH 7.2) under dark conditions. To initiate the photoreaction, the samples was irradiated with 410 nm, and both the spectrum was collected. This process was repeated until there was no change in the spectrum. The kinetics of photocleavage was studied by plotting absorbance versus time to obtain the profile which was further fitter to one phase decay equation to get half-life and rate constant value:

$$y = (y_0 - y_{max})e^{-kx} + y_{max}$$

where y_0 is initial absorbance at λ_{max}; y_{max} is absorbance at saturation (or maximum conversion); and k is the rate constant.

3. Homology modeling for human CAV

Using the SwissModel program, a homology model for hCA-V was made to provide a more accurate protein working model for structural comparison and molecular docking. The User Template method was used through the SwissModel program which uses a target sequence for hCA-V and the coordinates of a template crystal structure to make the homology model. Structural information is taken from the template crystal structure and used to create the homology model in regions where the sequences of the template and the target are identical. Areas of sequence misalignments are solved using structural databases, statistical potentials, and energy minimization through the ProMod3 comparative modeling engine in the SwissModel software. Homology models were made using various templates. Model 1 was formed using template 1CA2 (carbonic anhydrase II), model 2 was formed using template 6FE2 (carbonic anhydrase IX), and model 3 was formed using template 1URT (murine carbonic anhydrase V). Model 3 was used for subsequent docking due to a higher similarity between murine CA-V and human CA-V. Model 3 (template PDB: 1URT) has a GMQE score of 0.72, a QMEAN Z-score of -0.75, with a sequence identity of 78.90%. The sequence identity is well above that recommended for a homology model (50%), and the GMQE score and QMEAN Z-score indicate average scores for quality of the structure.

4. Activity with bCA – NPA assay using UV/vis spectroscopy

The effects of probe binding with bCA-II before and after photocleaving reaction was studied by monitoring the esterase activity of bCA-II in the presence of *p*-nitrophenyl acetate (NPA) as the substrate. As a result of enzymatic activity, NPA is hydrolyzed to give *p*-nitrophenolate ion, which has a λ_{max} at 400nm. Hence, the enzymatic activity in the absence and presence of probe can be determined spectroscopically by monitoring the increase in absorbance with time. In this study, a stock solution of 2 mg/mL (~70 μM) protein in buffer, 10 mM probe in DMSO and 125 mM NPA in dry acetonitrile were prepared. During analysis, the enzyme, probe and NPA concentrations were kept constant at 2 μM, 2 μM and 250 μM respectively. The absorbance was recorded at 400 nm every 60 sec for up to 10 minutes and was plotted with respect to time. To study the effect of photoirradiation on the enzymatic activity, the solution of

probe in buffer was irradiated with 410 nm for 20 minutes before the addition of protein and NPA. All the studies were performed in the absence of light at room temperature.

5. IC_{50} Determination

IC_{50}s for **PCE**, **PCEM**, **dPCE**, **dPCEM**, and ethoxzolamide were determined using a spectrophotometric assay with NPA as the substrate, where hydrolysis of NPA by bCA-II is measured kinetically at 405 nm. Assays were performed in four or more replicates in a 96-well plate format, with a final assay volume of 200 µL per well. Inhibitor solutions were prepared *via* 4-fold serial dilution, with final inhibitor concentrations ranging from 200 µM to 0.000763 µM (10 different inhibitor concentrations total). DMSO was kept at a constant 2% concentration for all wells. Each well contained 500 nM bCA-II, inhibitor, and NPA (where 75 µL of a 200 µM NPA stock was diluted to give a final assay volume of 200 µL and a final NPA concentration of 75 µM, added last to initiate the reaction). Inhibitor and bCA-II were pre-incubated for 15 min. Reactions were initiated by the addition of NPA and read immediately. Absorbance at 405 nm was monitored continuously for 30 minutes. The initial rates were calculated from the linear portion of the absorbance-time curves, from 800 s to 1800 s. Data were normalized to a no-inhibitor control and fitted to a [inhibitor] vs. response (three parameter) equation using GraphPad Prism to determine IC_{50} values.

6. MTT assay

The cytotoxic effect of **PCE** and **PCEM** on HepG2 cells in both protected and deprotected form was assessed using the MTT Cell Proliferation Colorimetric Assay Kit. Initially, cells were seeded in a 96-well plate at a density of 4000 cells per well using DMEM media. After 24 hours, varying concentrations of **PCE**, **dPCE**, **PCEM**, and **dPCEM** (deprotection formed via photoirradiation at 410 nm) were introduced to achieve a final volume of 150 µL media per well. A control group of wells containing an equivalent volume of DMSO served as reference. Following a 24-hour incubation period, media were removed, and cells were rinsed with LCIS (Live Cell Imaging Solution). Subsequently, 100 µL of MTT solution was added to each well, followed by a 2-hour incubation at 37°C. Finally, 100 µL of DMSO was added to dissolve the precipitates, followed by a 10-minute agitation. Absorbance readings at 490 nm were taken using a plate reader and normalized against the absorbance of control wells treated with DMSO alone.

7. Confocal imaging

Cells were seeded in a 4-well IBIDI dish at a density of 4000 cells per well in 700 µL of growth media. The following day, cells underwent PBS buffer washing, after which 700 µL of 1 µM **PCE** and **PCEM** and of 0.1 µM mitotracker prepared in Live Cell Imaging Solution (LCIS), were added and allowed to incubate for 20 minutes. Subsequently, cells were washed with LCIS and directly imaged under a Zeiss 710 Laser Scanning confocal microscope using an excitation/emission of 560/580-700 nm for mitotracker dye and 405/450-530 nm for detecting probe **PCE** and **PCEM**.

7. Co-localization analysis

Images were processed in Fiji[1] to remove background fluorescence, then the Colocalization Finder plugin was used to evaluate Pearson's Correlation Coefficient (Pearson's R) for each cell, with the threshold set to exclude fluorescence within the nucleus of each cell (intensity

minimum ≥1). An unpaired t-test was performed to determine the significance of difference between **PCEM** and **PCE** treated cells.

8. Citrulline assay:

HepG2 cells were seeded in a T-75 flask with growth media containing phenol red. After 48 hours, the cells were washed with PBS and treated with 50 μM **PCE**, **PCEM**, or DMSO solutions, along with 50 μM ornithine in fluorobrite media. The cells were then incubated for 2 hours. Subsequently, they were irradiated with 410 nm light for 5 minutes and incubated at 37°C overnight.

For mitochondrial extraction, a cell suspension of 9.5 × 10^6 cells/mL was prepared in lysis buffer, keeping the temperature at 4°C using an ice bath. The chilled suspension was homogenized with a Dounce homogenizer until 90% lysis was confirmed microscopically. The lysate was diluted to 10 mL by adding 9 mL of 1x separation buffer. Then, 50 μL of Anti-TOM22 microbeads were added to selectively bind the mitochondria. The mixture was incubated at 4°C for 1 hour with gentle shaking. The solution was then passed through an LS column, which was washed with 1x separation buffer. Finally, the mitochondria were eluted by pressing the plunger firmly into the column using 1x separation buffer.

Then citrulline concentrations was determined using a colorimetric assay. 50 μL of each mitochondria solution was added to 2 mL screwcap tubes, followed by the addition of 5 μL of SDS solution and 5 μL of Proteinase K solution. The mixtures were incubated at 37°C for 2 hours. Post-incubation, 250 μL of Assay Reagent A and 50 μL of Assay Reagent B were added, and the tubes were incubated at 95°C for 30 minutes. After cooling the tubes to 4°C, samples were centrifuged at 18,000 x g for 10 minutes. The supernatant (200 μL) was transferred to a 96-well plate, and absorbance was measured at 550 nm.

9. Intracellular pH assay:

Cell Culture and Sample Preparation

HeLa cells were seeded in two 24-well tissue culture plates at a density of 0.05 x 10^6 cells per well in 0.5 mL of growth medium. The following day, cells were washed with 2 x 0.5 mL PBS, then incubated with 0.5 mL pH RhodoRed Solution (1 μL pHRhodo Red + 10 μL PowerLoad diluted in 17 mL FluoroBrite DMEM) for 30 minutes at 37 ºC, 5% CO_2. Cells were washed with 2 x 0.5 mL PBS, then incubated with **PCE**, **PCEM**, ethoxzolamide (50 μM) or DMSO (0.5%) for 30 minutes at 37 ºC, 5% CO_2. Cells were further washed with 2 x 0.5 mL PBS, then placed in 0.5 mL FluoroBrite DMEM. Then, **dPCE** and **dPCEM** samples were irradiated with 410 nm light for 5 minutes, one well at a time, at room temperature, and all samples were incubated for a further 30 minutes at 37ºC, 5% CO_2. The 0.5 mL medium was removed and the cells were detached in 0.2 mL growth medium with 75 μL EDTA solution (12.5 mM EDTA, pH 6.5, final [EDTA] 3.4 mM) during a 45 minute incubation period at 37 ºC, 5% CO_2. At this point, each well was diluted up to 1.0 mL with either cellular pH calibration buffers (4.5, 5.5, 6.5, 7.5) containing valinomycin and nigericin (10 μM) or with FluoroBrite DMEM, all pre-warmed to 37 ºC. The pH of each calibration curve well was verified using a pH probe immediately after adding each pH buffer.

Calibration Curve

Cells were incubated in cellular pH calibration buffers (pre-warmed 37 ºC) for 10 minutes at room temperature to allow equilibration with the buffer solution before measurement. A Cytek Northern Lights Spectral Flow Cytometer was used to record cellular fluorescence intensity at 581 nm. Each acquisition measured 3,000 cells after gating, and median fluorescence was plotted with respect to pH to obtain the calibration curve. This curve was generated for each individual experiment and used to calculate the pH over time of all subsequent samples in the experiment.

Response to perturbations in CO_2

Cells were incubated in FluoroBrite DMEM for at least 15 minutes at room temperature to allow equilibration with the buffer solution before measurement. A solution of saturated aqueous CO_2 gas was generated by bubbling CO_2 gas in MilliQ water at 4 ºC over a period of 1.5 hours. This solution was then aliquoted into multiple Eppendorfs and kept on ice to ensure consistency of CO_2 concentration throughout the duration of the experiment. For each treatment well, fluorescence intensity at 581 nm was measured continuously over the course of 2.5 minutes (150 seconds). The baseline reading of cells was measured for 20 seconds before 65 µL of the sat. aq. CO_2 solution was added, followed by a further 15 seconds of mixing of the well and then sustained measurement for the remaining time.

Analysis

The data, gated for single cells, was processed to find the median cellular pH over time for each well. This was accomplished by binning the data over time (10 total bins), converting fluorescence values to pH using the calibration curve for the experiment, and calculating the running median using the Python library numpy. The pH values for the bins at ~33 sec and ~66 sec were used to calculate a slope, representative of the cellular response to perturbations in CO_2. A one-way ANOVA was performed to assess statistical significance of the difference of slopes between control and experimental populations.

All-Atom Contacts	Clashscore, all atoms:	0.53		99th percentile* (N=1784, all resolutions)
	Clashscore is the number of serious steric overlaps (> 0.4 Å) per 1000 atoms.			
Protein Geometry	Poor rotamers	12	5.91%	Goal: <0.3%
	Favored rotamers	185	91.13%	Goal: >98%
	Ramachandran outliers	1	0.43%	Goal: <0.05%
	Ramachandran favored	219	93.59%	Goal: >98%
	MolProbity score^	1.69		90th percentile* (N=27675, 0Å - 99Å)
	Cβ deviations >0.25Å	2	0.90%	Goal: 0
	Bad bonds:	0 / 1962	0.00%	Goal: 0%
	Bad angles:	16 / 2678	0.60%	Goal: <0.1%
Peptide Omegas	Cis Prolines:	2 / 15	13.33%	Expected: ≤1 per chain, or ≤5%
Additional validations	Chiral volume outliers	0/284		
	Waters with clashes	0/0	0.00%	See UnDowser table for details

[illegible]

Figure S1: Quality assessment of Model 3 homology structure of hCA-VA (template PDB: 1URT) from Duke University Molprobity all-atom structure assessment software. Rotamer and angle quality is low in this model but mostly apparent in regions other than the active site. The relatively well conserved active site provided confidence that this model can be used for docking.

SwissModel Assessment Score information:
GMQE: global model quality estimate, how accurate the structure is (scale of 1)
QMEAN Z-score: how similar model is to experimental structures (needs to score above -4)

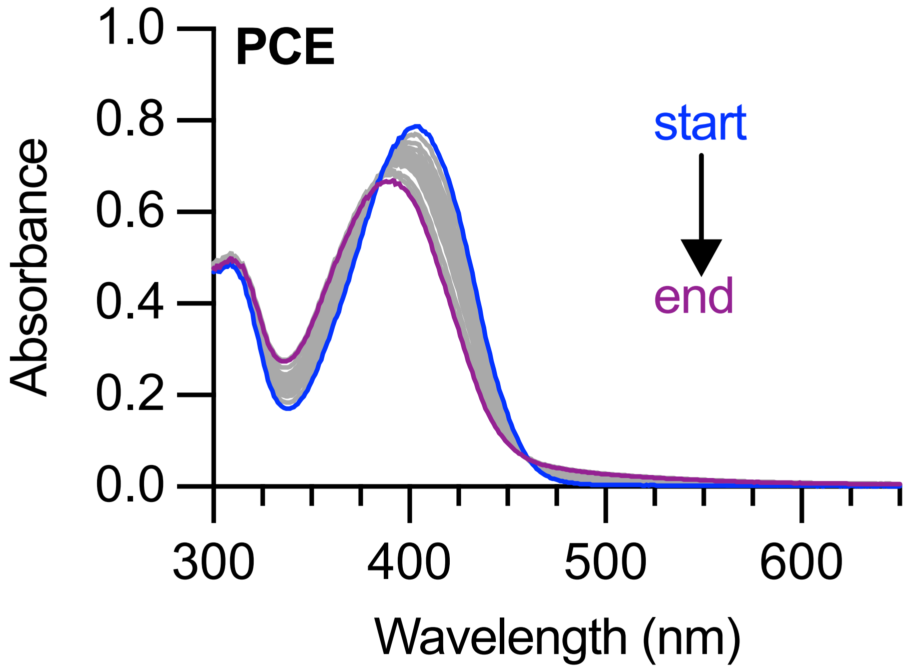

Figure S2. Changes in absorbance of **PCE** following irradiation with 410 nm light.

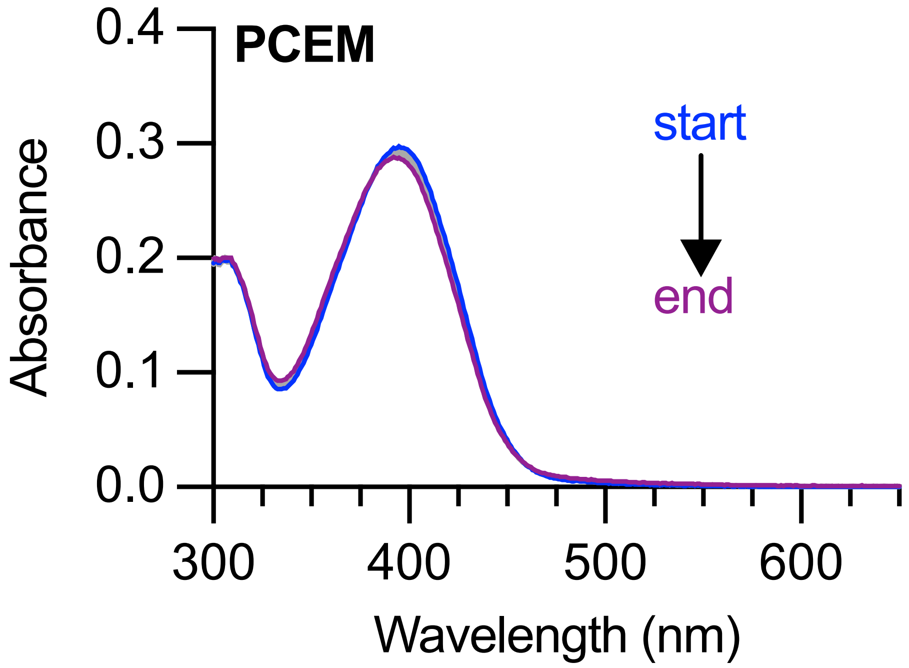

Figure S3. Changes in absorbance of **PCEM** following irradiation with 410 nm light.

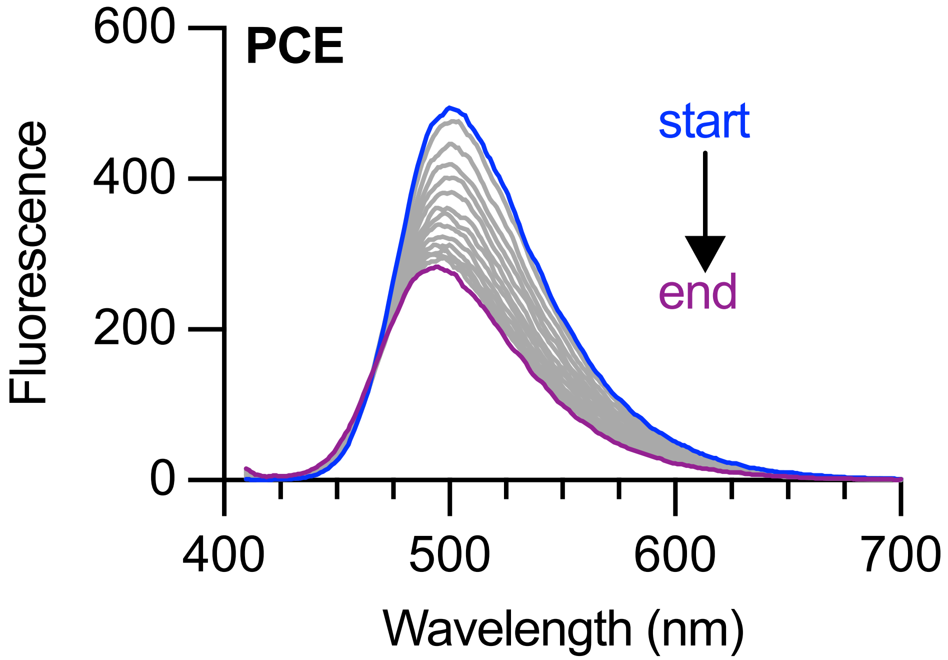

Figure S4. Changes in fluorescence of **PCE** following irradiation with 410 nm light. Excitation wavelength 390 nm.

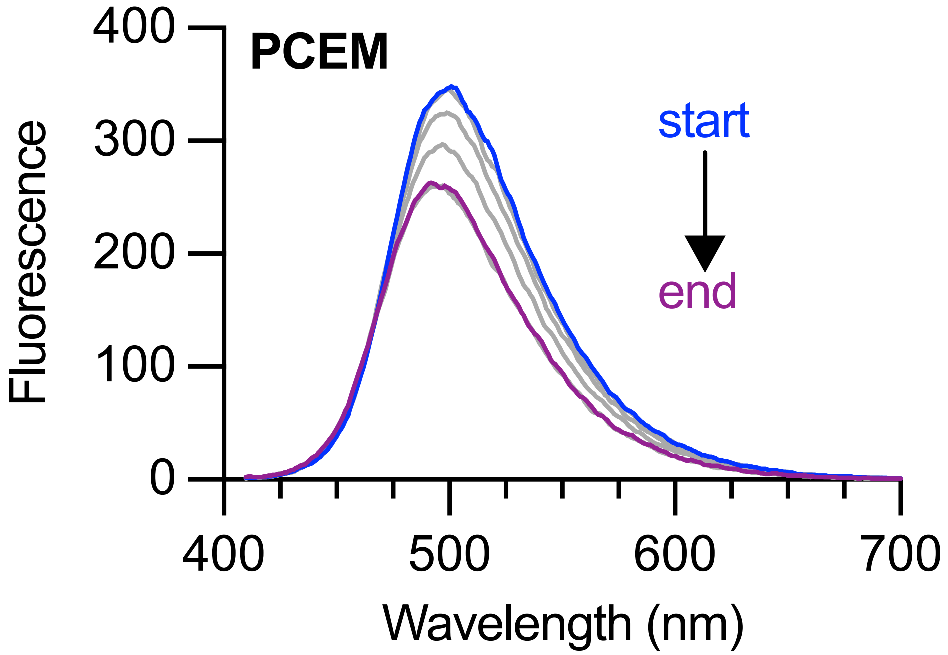

Figure S5. Changes in fluorescence of **PCEM** following irradiation with 410 nm light. Excitation wavelength 390 nm.

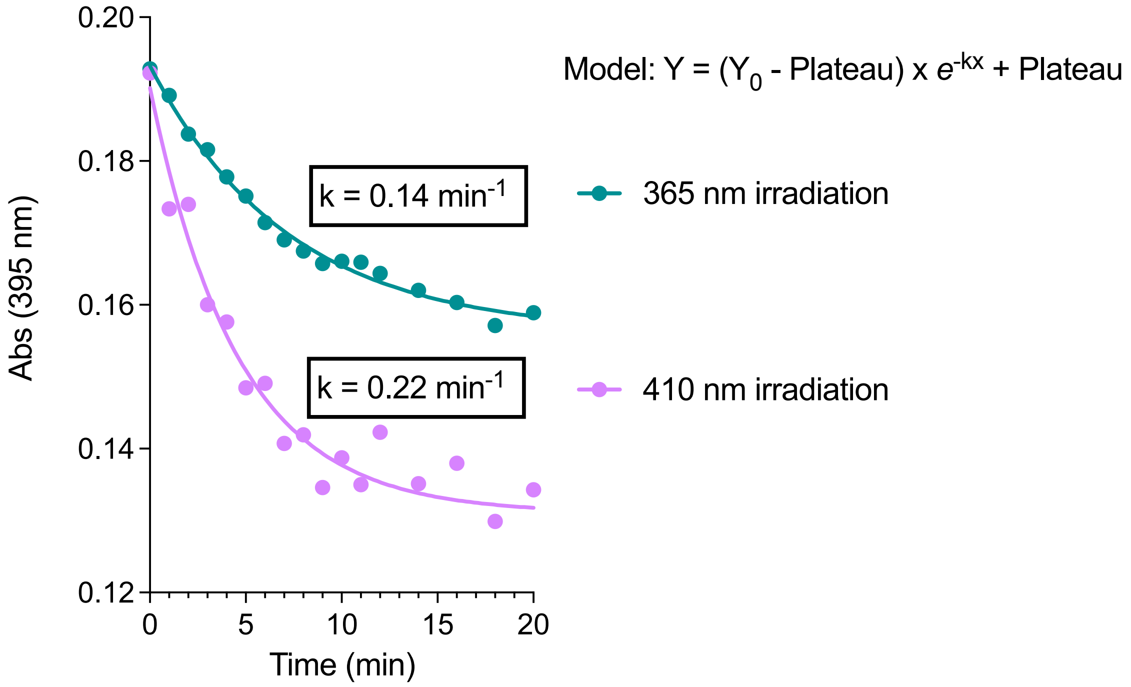

Figure S6. Kinetics of **PCE** deprotection as monitored by UV-vis spectroscopy.

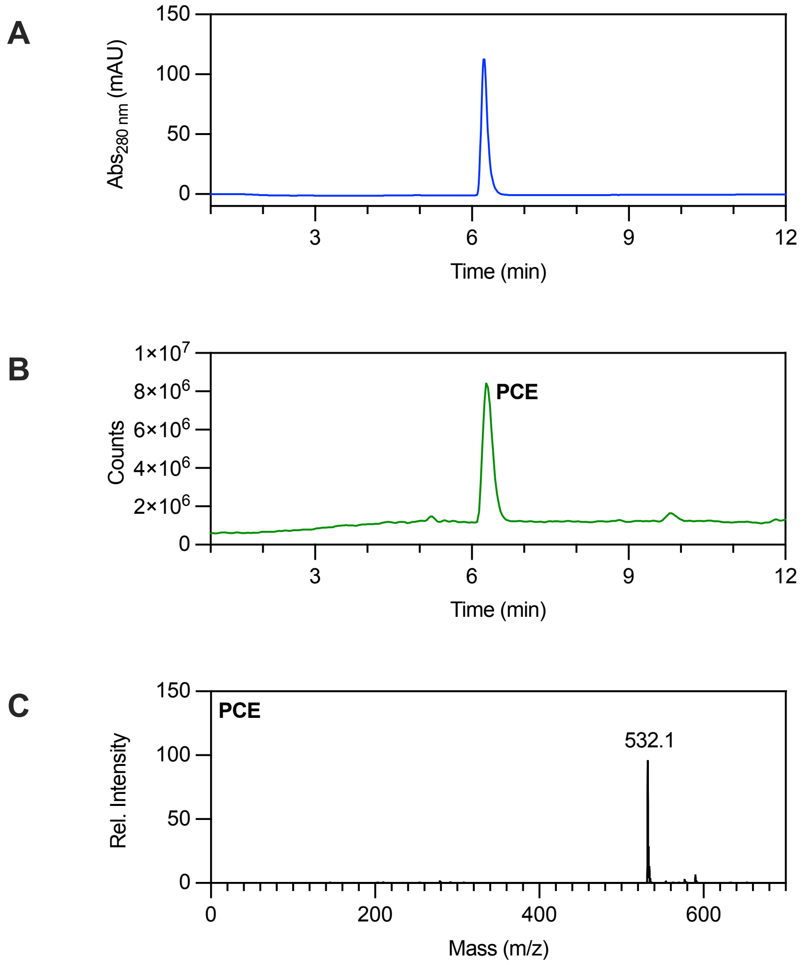

Figure S7. LC/MS analysis of **PCE** prior to irradiation. A) Absorbance trace (280 nm); B) Mass spectral trace, ESI positive mode; C) Mass spectrum for peak labeled as “**PCE**.”

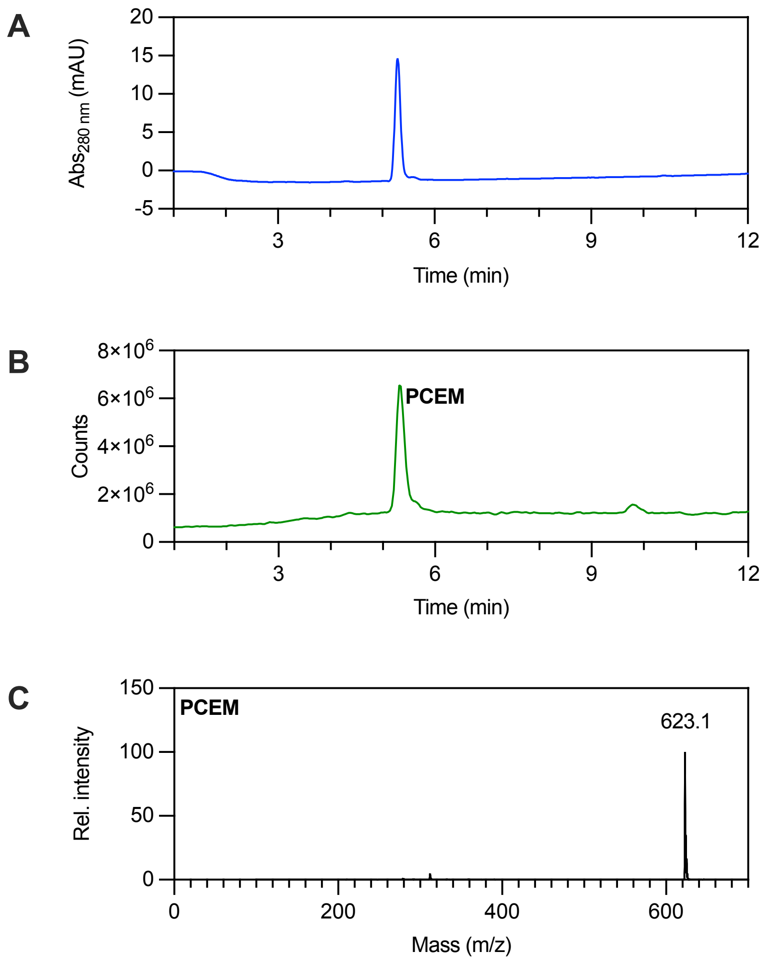

Figure S8. LC/MS analysis of **PCEM** prior to irradiation.

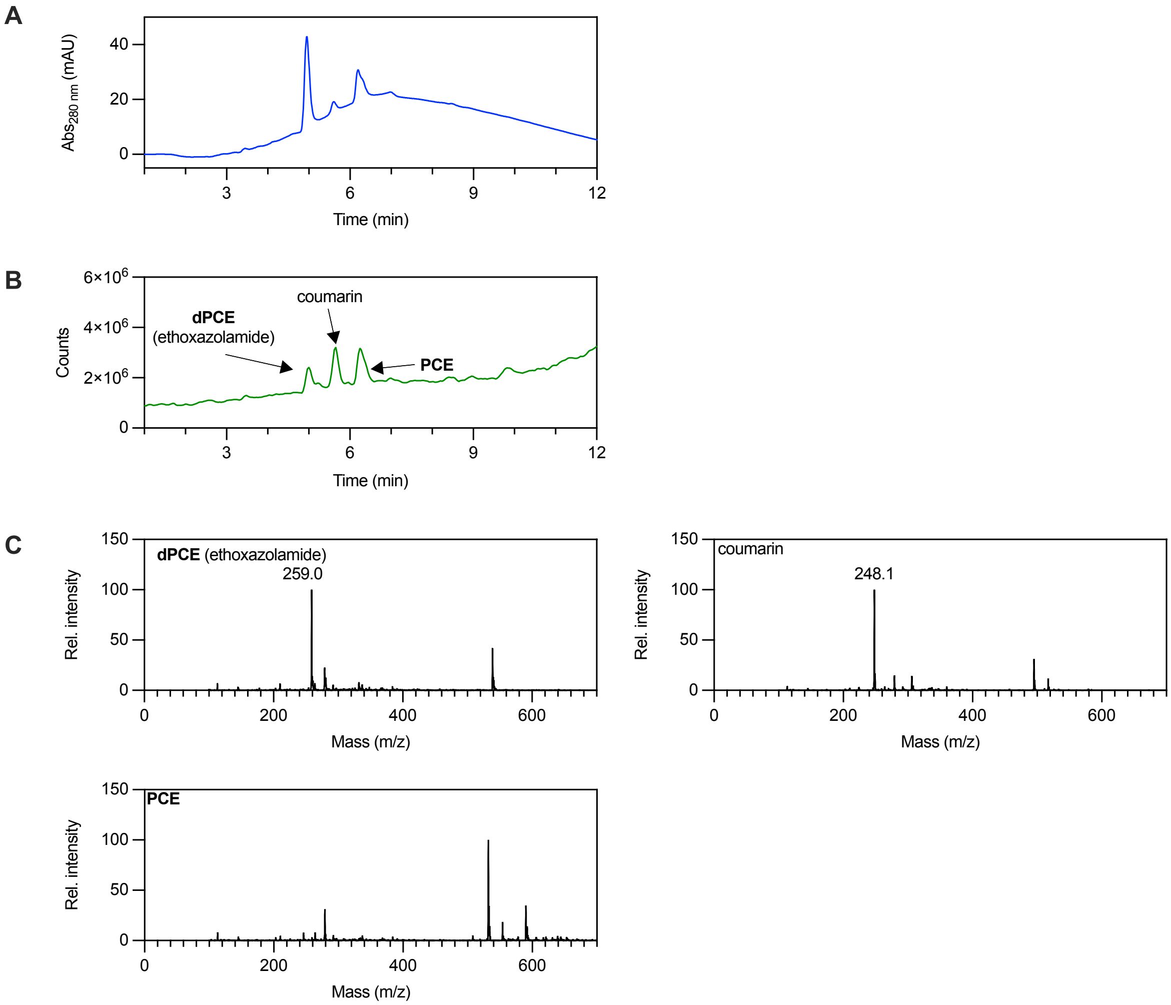

Figure S9. LC/MS analysis of **PCE** following irradiation. A solution of **PCE** was irradiated with 410 nm light to achieve partial conversion to coumarin and **dPCE** (ethoxzolamide).

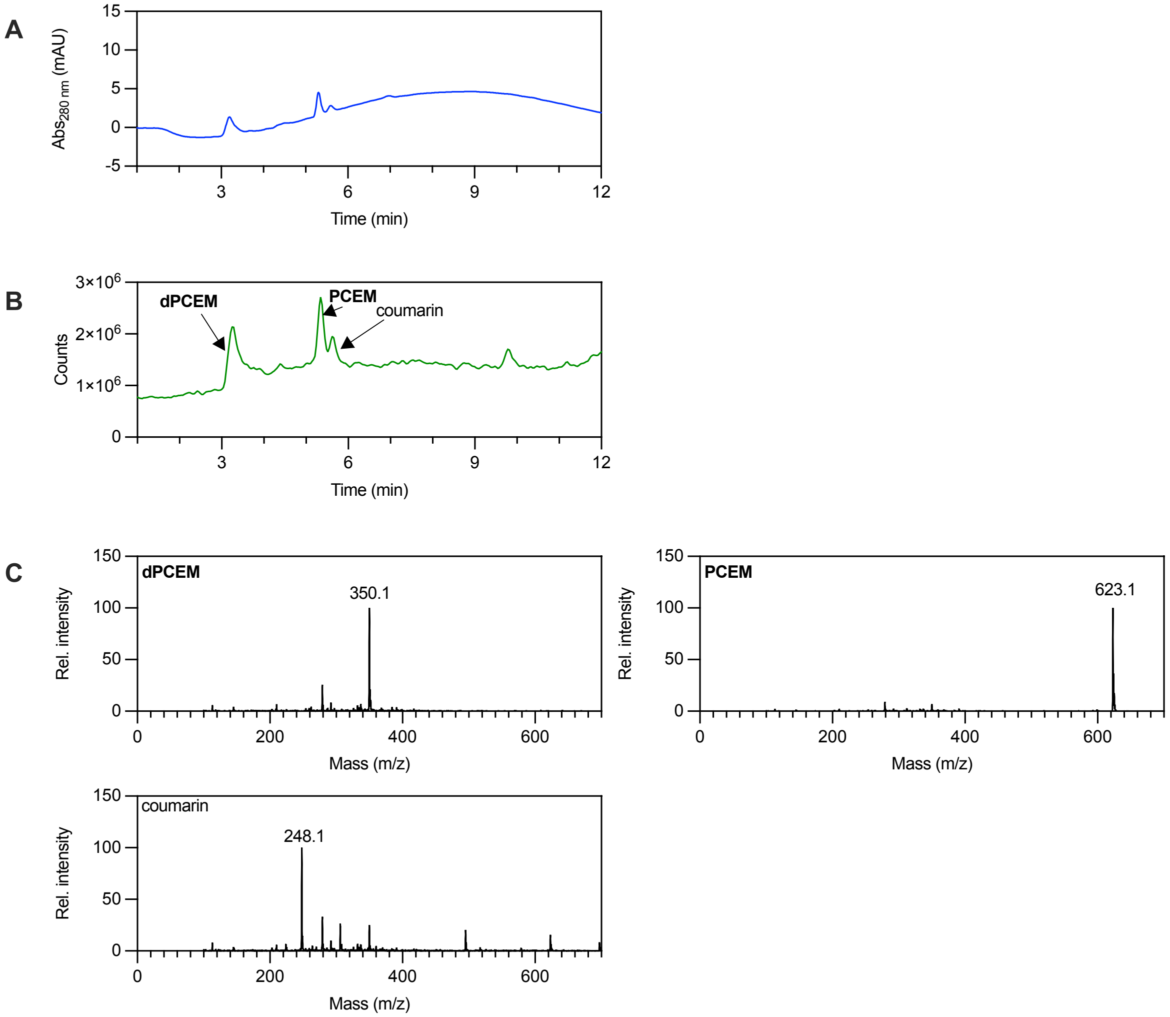

Figure S10. LC/MS analysis of **PCEM** following irradiation. A solution of **PCEM** was irradiated with 410 nm light to achieve partial conversion to coumarin and **dPCEM**.

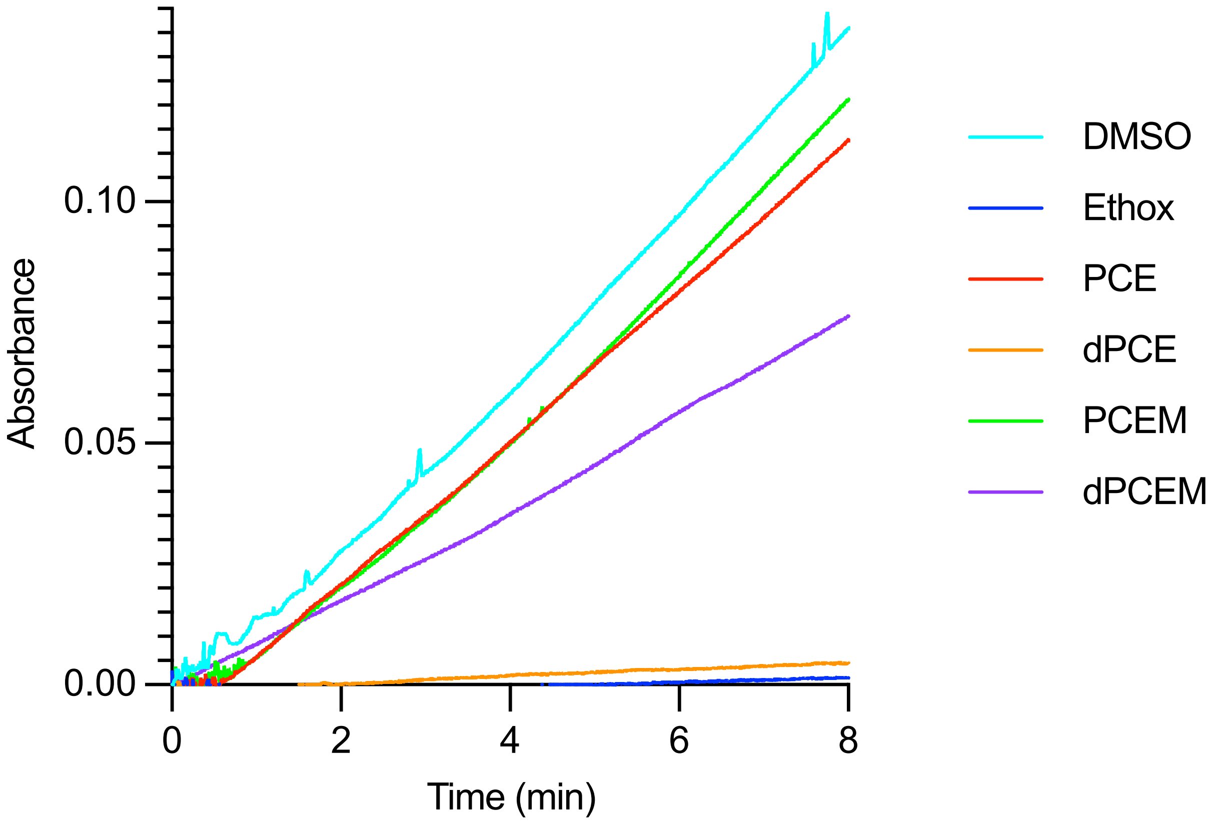

Figure S11. Nitrophenylacetate (NPA) assay data. Absorbance at 400 nm was tracked for solutions of 2 μM bCA and 250 μM NPA in the presence of 2 μM of the indicated molecule.

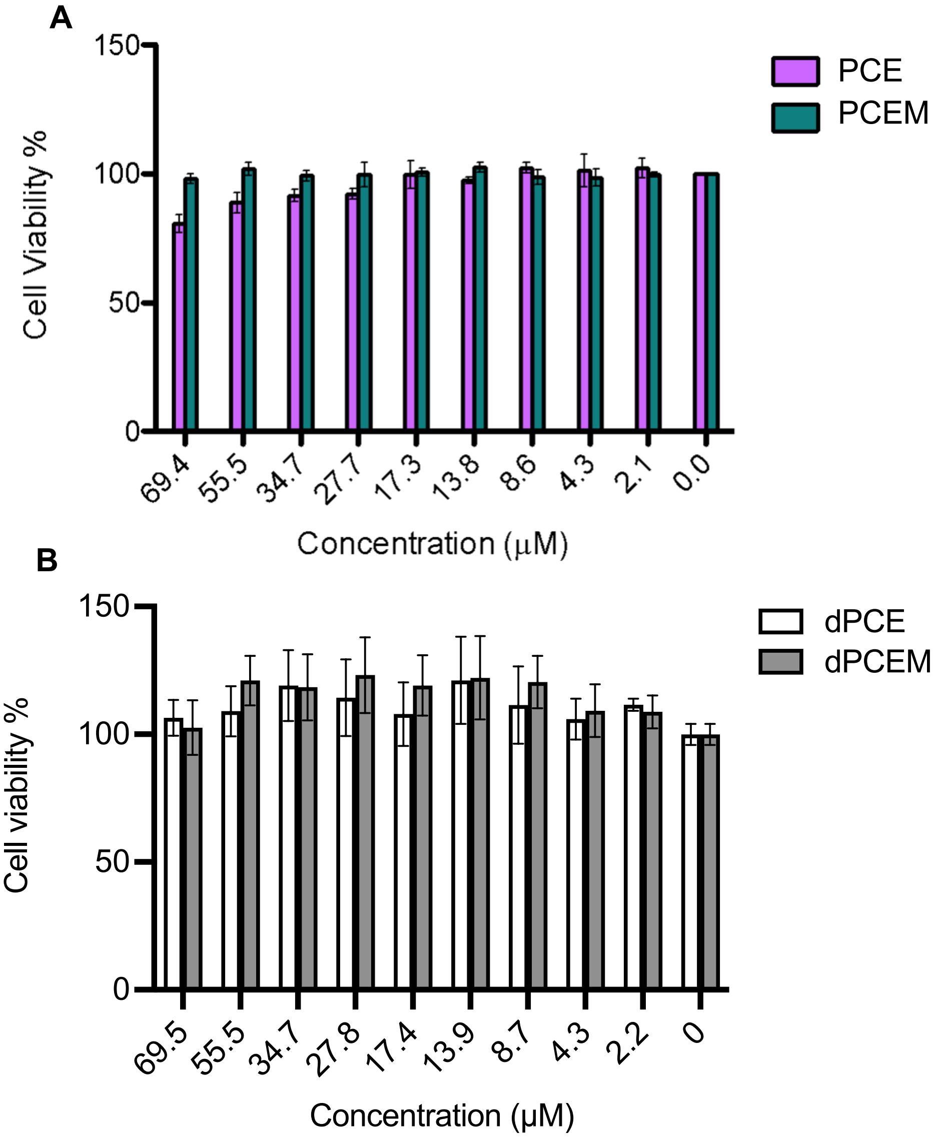

Figure S12. MTT assay to assess cytotoxicity of A) **PCE** (pink) and **PCEM** (turquoise) and B) **dPCE** (white) and **dPCEM** (grey) towards HepG2 cells.

Note: An increase in cell viability relative to control samples was observed in certain **dPCE** and **dPCEM** samples. One potential contributing factor to this effect may be the release of the coumarin derivative during photodeprotection, and this molecule may possess antioxidant properties. These antioxidant effects could enhance the reduction of MTT to formazan either directly or indirectly, thereby leading to elevated cell viability readings.[2–4] It is unlikely that the deprotection products are promoting cellular proliferation, as both coumarins and sulfonamides have been reported to exhibit anti-proliferative activity.

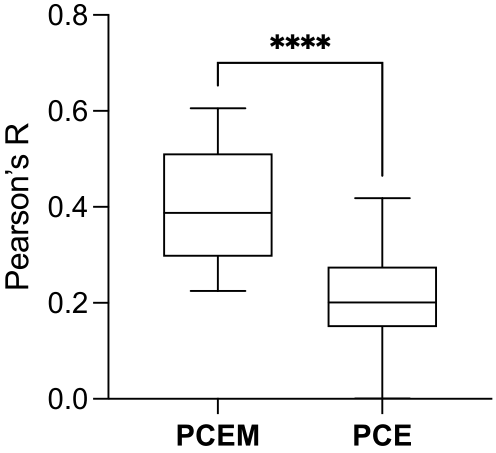

Figure S13. Pearson's Correlation Coefficient (Pearson's R) analysis of **PCE** and **PCEM** with MitoTracker Red. Groups were analyzed for statistical difference using an unpaired t test ($p < 0.0001$).

References

1 J. Schindelin, I. Arganda-Carreras, E. Frise, V. Kaynig, M. Longair, T. Pietzsch, S. Preibisch, C. Rueden, S. Saalfeld, B. Schmid, J.-Y. Tinevez, D. J. White, V. Hartenstein, K. Eliceiri, P. Tomancak and A. Cardona, *Nat Methods*, 2012, **9**, 676–682.

2 D. Zúñiga-Núñez, P. Barrias, G. Cárdenas-Jirón, M. S. Ureta-Zañartu, C. Lopez-Alarcón, F. E. Morán Vieyra, C. D. Borsarelli, E. I. Alarcon and A. Aspée, *RSC Adv.*, 2018, **8**, 1927–1933.

3 T. P. N. Talorete, M. Bouaziz, S. Sayadi and H. Isoda, *Cytotechnology*, 2006, **52**, 189–198.

4 I. Kostova, S. Bhatia, P. Grigorov, S. Balkansky, V. S. Parmar, A. K. Prasad and L. Saso, *Curr Med Chem*, 2011, **18**, 3929–3951.

Compound 1:

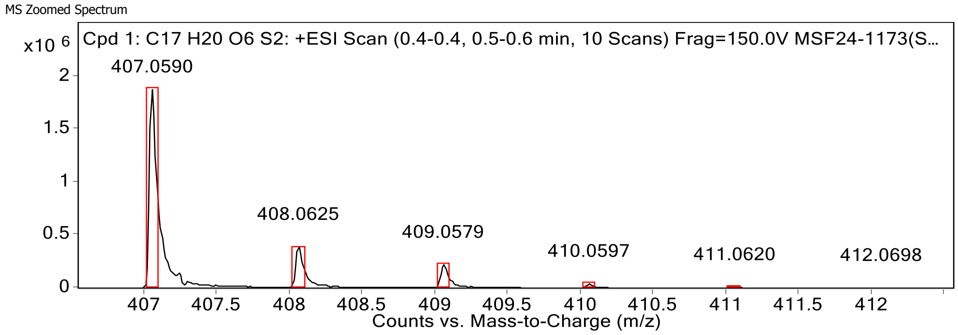

MS Spectrum Peak List

Obs. m/z	Calc. m/z	Charge	Abundance	Formula	Ion Species	Tgt Mass Error (ppm)
407.0590	407.0594	1	1882222	C17H20O6S2	(M+Na)+	0.96
408.0625	408.0624	1	390188	C17H20O6S2	(M+Na)+	-0.2
409.0579	409.0577	1	220662	C17H20O6S2	(M+Na)+	-0.58
410.0597	410.0599	1	40662	C17H20O6S2	(M+Na)+	0.71
411.0620	411.0574	1	9185	C17H20O6S2	(M+Na)+	-11.16

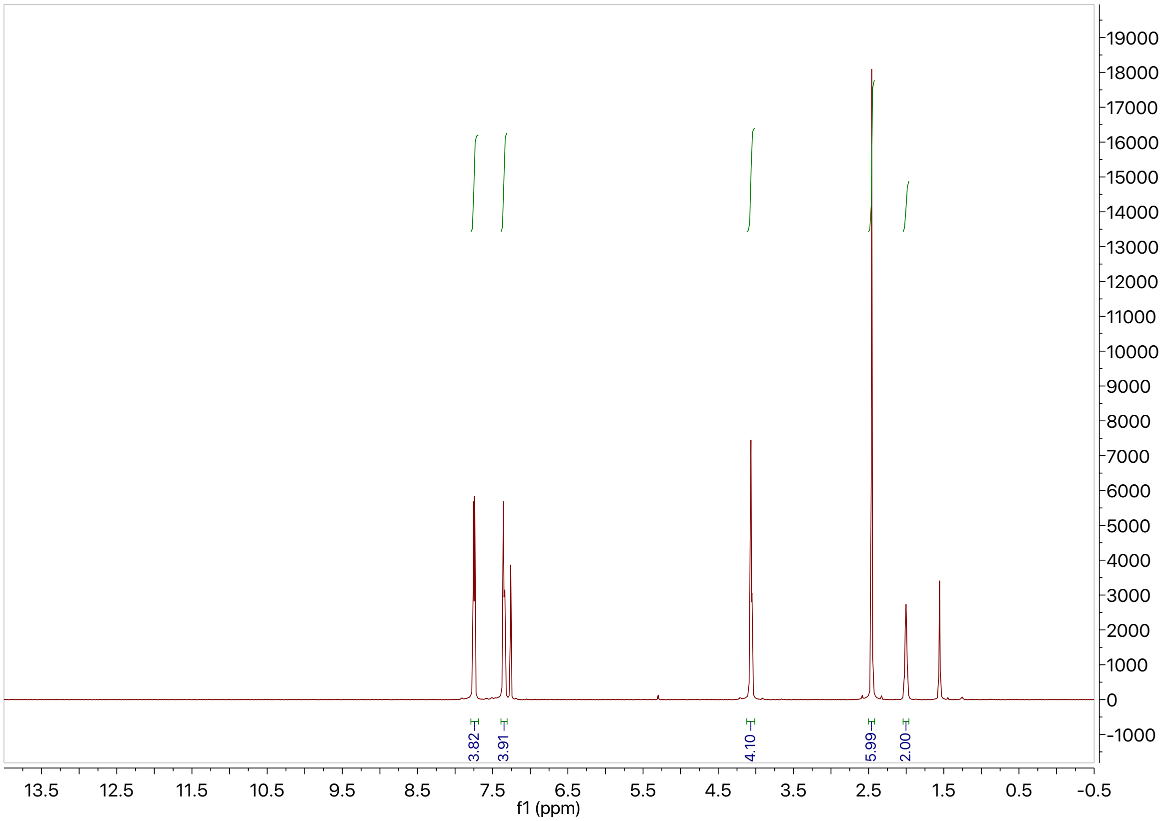

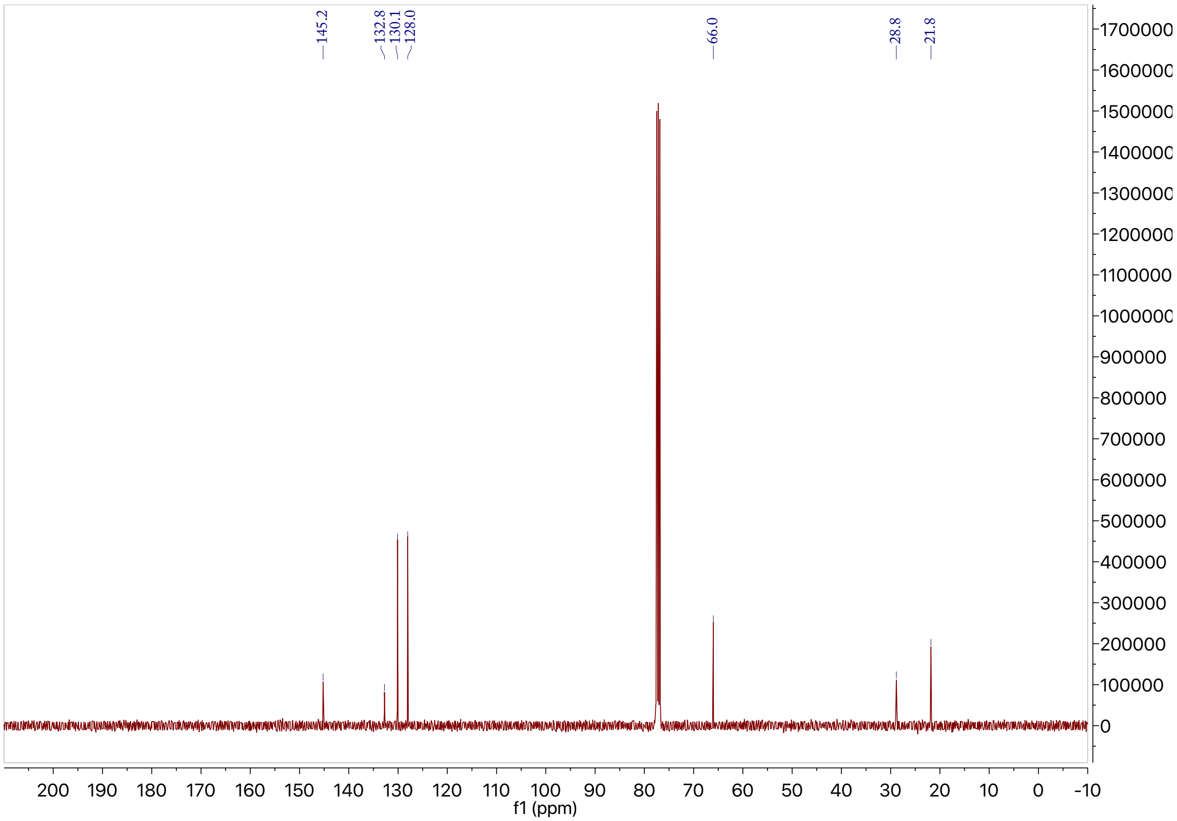

145.2
132.8
130.1
128.0
66.0
28.8
21.8
1700000
1600000
1500000
1400000
1300000
1200000
1100000
1000000
900000
800000
700000
600000
500000
400000
300000
200000
100000
0
200 190 180 170 160 150 140 130 120 110 100 90 80 70 60 50 40 30 20 10 0 -10
f1 (ppm)

Compound 2:

MS Zoomed Spectrum

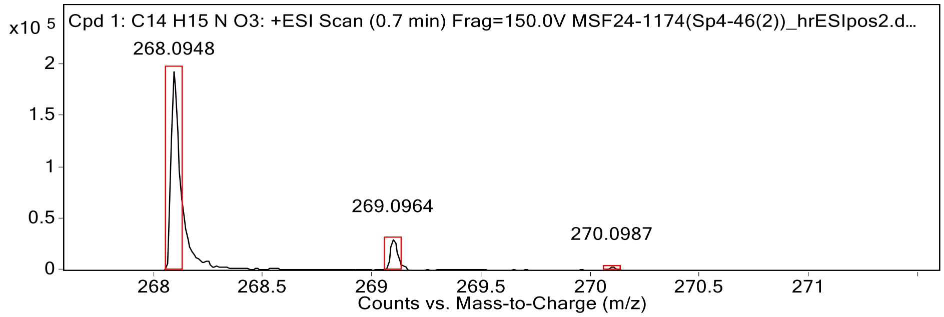

MS Spectrum Peak List

Obs. m/z	Calc. m/z	Charge	Abundance	Formula	Ion Species	Tgt Mass Error (ppm)
268.0948	268.0944	1	197616	C14H15NO3	(M+Na)+	-1.29
269.0964	269.0977	1	30451	C14H15NO3	(M+Na)+	4.55
270.0987	270.1001	1	3384	C14H15NO3	(M+Na)+	5.14
577.2525			6944217			

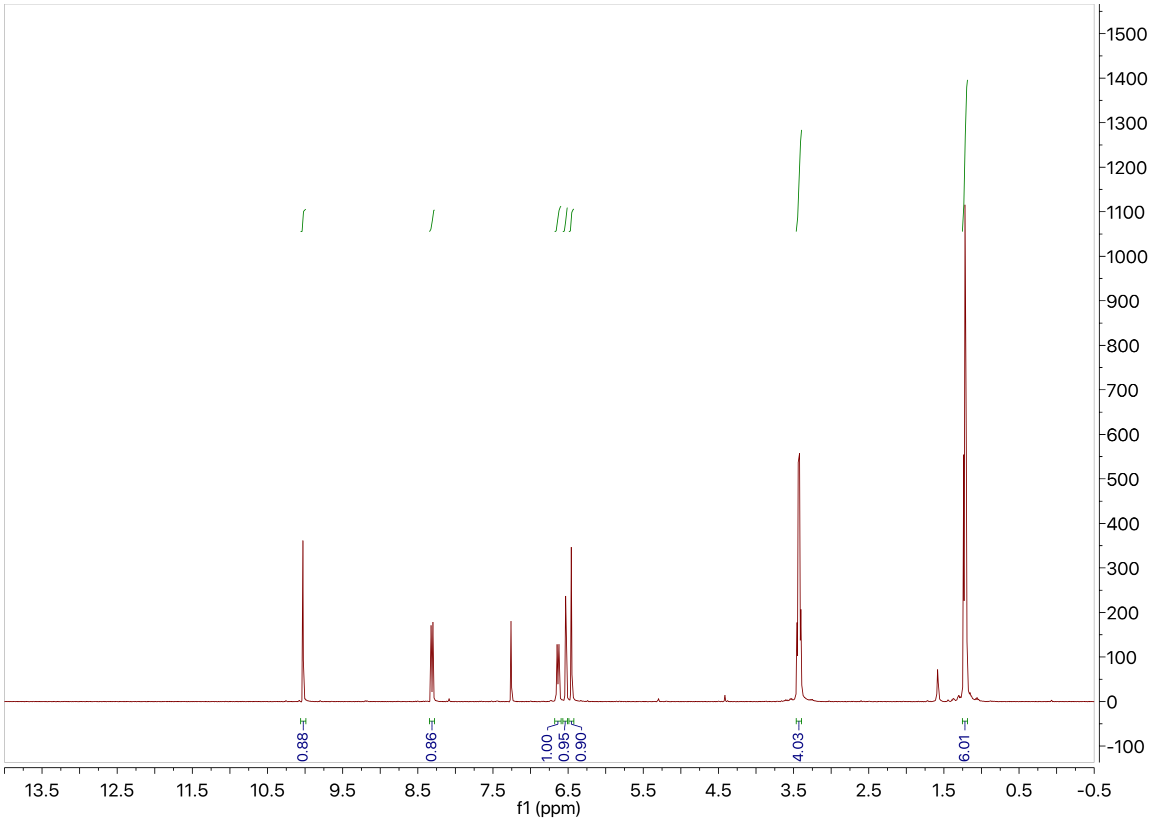

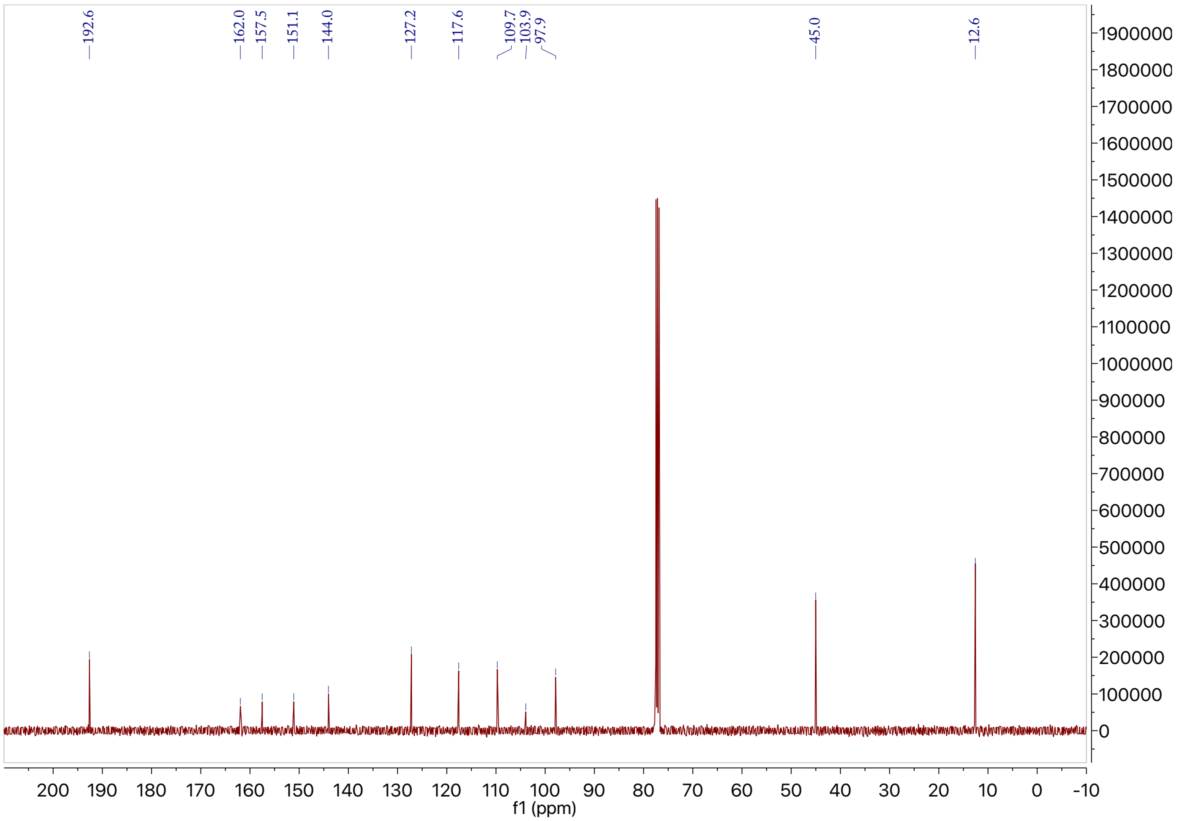
192.6
162.0
157.5
151.1
144.0
127.2
117.6
109.7
103.9
97.9
45.0
12.6
f1 (ppm)

Compound 3:

MS Zoomed Spectrum

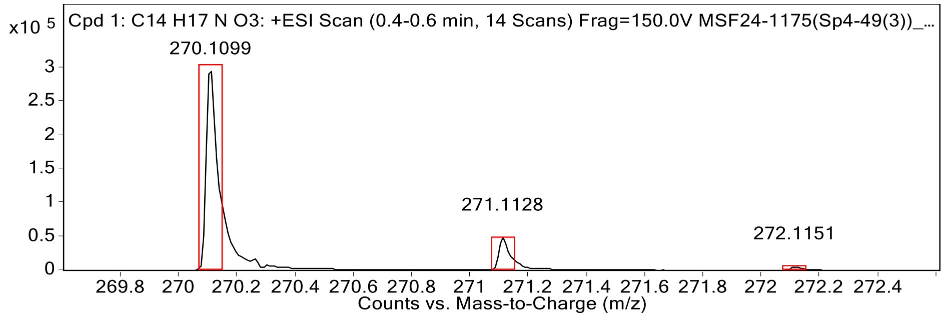

MS Spectrum Peak List

Obs. m/z	Calc. m/z	Charge	Abundance	Formula	Ion Species	Tgt Mass Error (ppm)
270.1099	270.1101	1	301942	C14H17NO3	(M+Na)+	0.51
271.1128	271.1133	1	48636	C14H17NO3	(M+Na)+	2.05
272.1151	272.1158	1	5961	C14H17NO3	(M+Na)+	2.49
273.1329	273.1184	1	747	C14H17NO3	(M+Na)+	-53.24
274.1281	274.1209	1	184	C14H17NO3	(M+Na)+	-26.25

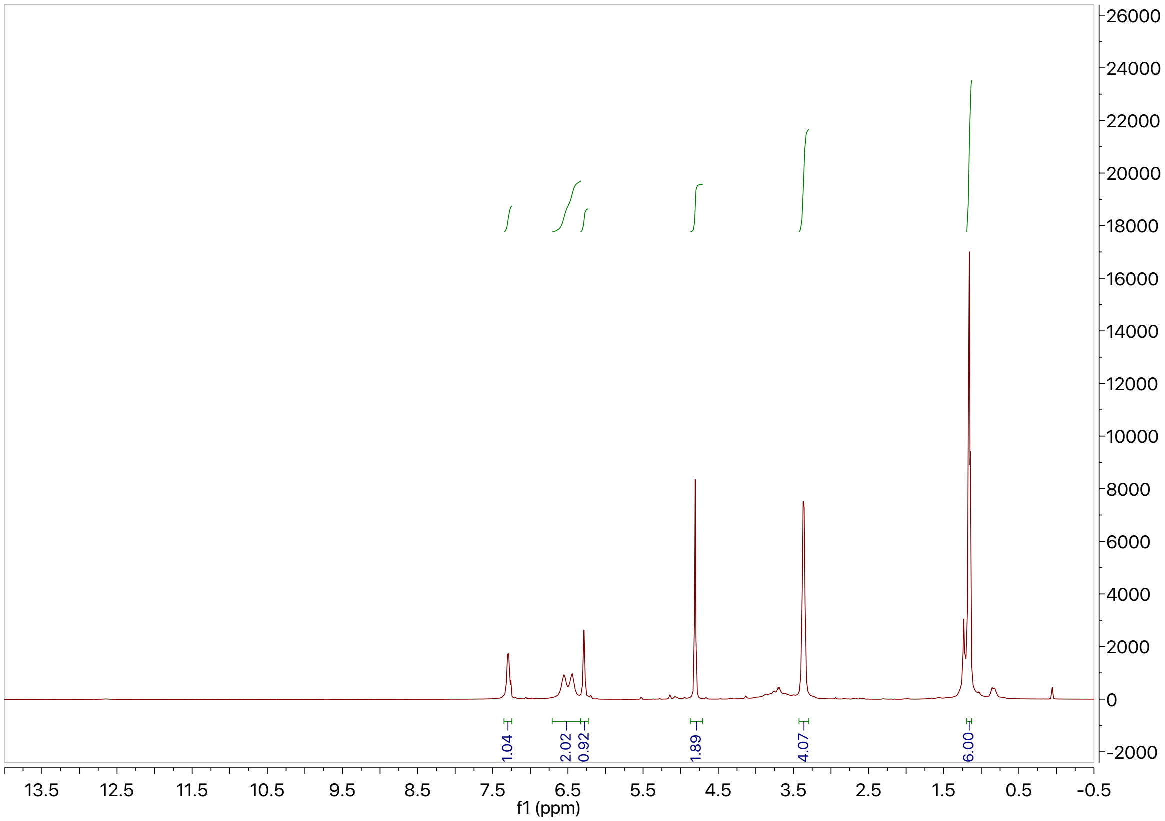

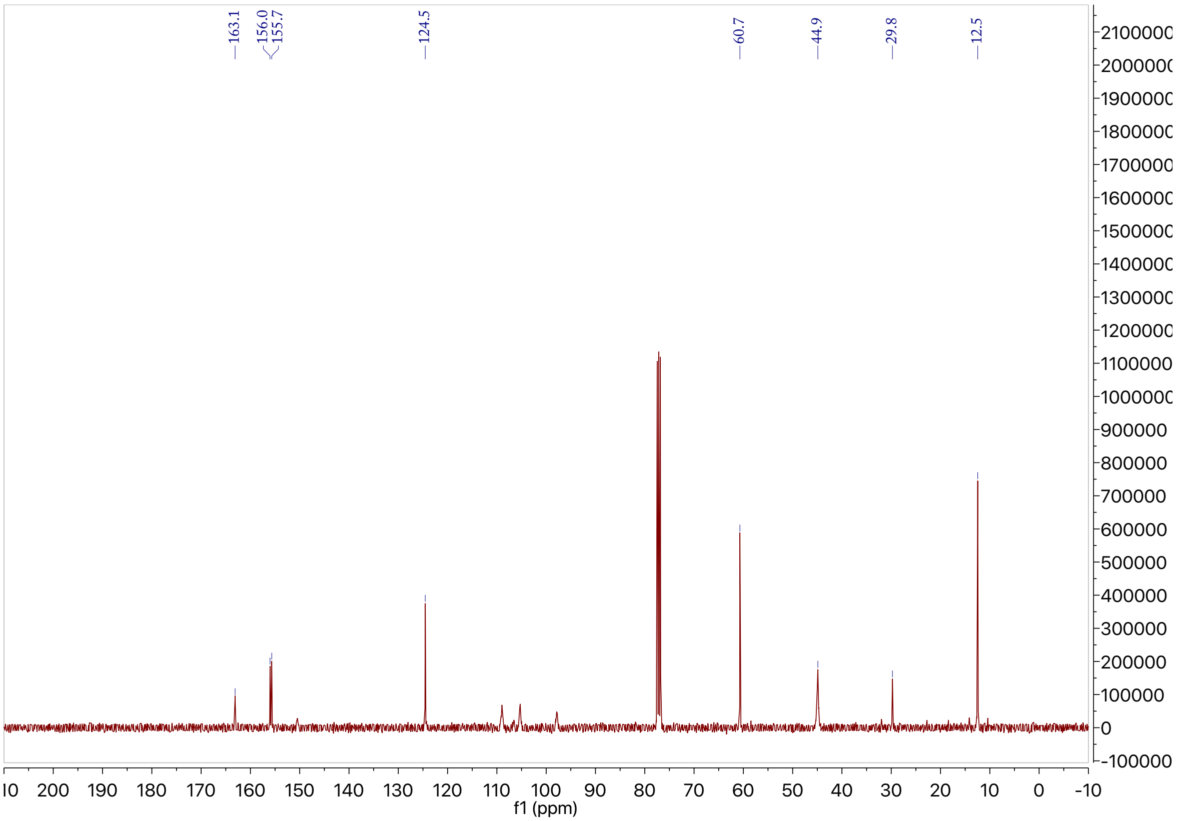
163.1
156.0
155.7
124.5
60.7
44.9
29.8
12.5
f1 (ppm)

Compound 4:

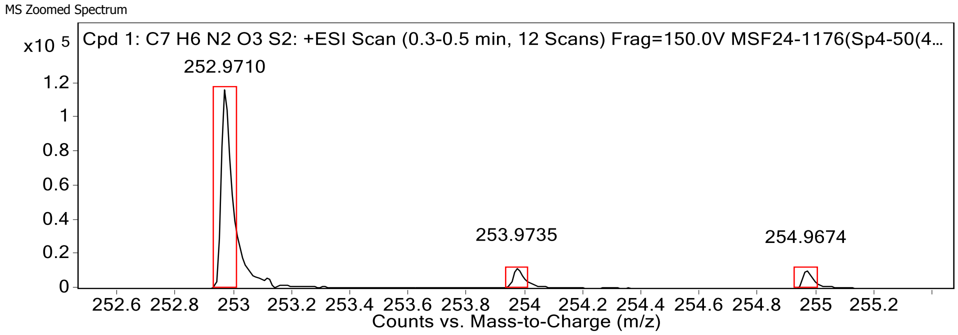

MS Spectrum Peak List

Obs. m/z	Calc. m/z	Charge	Abundance	Formula	Ion Species	Tgt Mass Error (ppm)
252.9710	252.9712	1	117634	C7H6N2O3S2	(M+Na)+	0.66
253.9735	253.9735	1	12069	C7H6N2O3S2	(M+Na)+	-0.14
254.9674	254.9679	1	10829	C7H6N2O3S2	(M+Na)+	2.19
255.9034	255.9702	1	78	C7H6N2O3S2	(M+Na)+	261.2

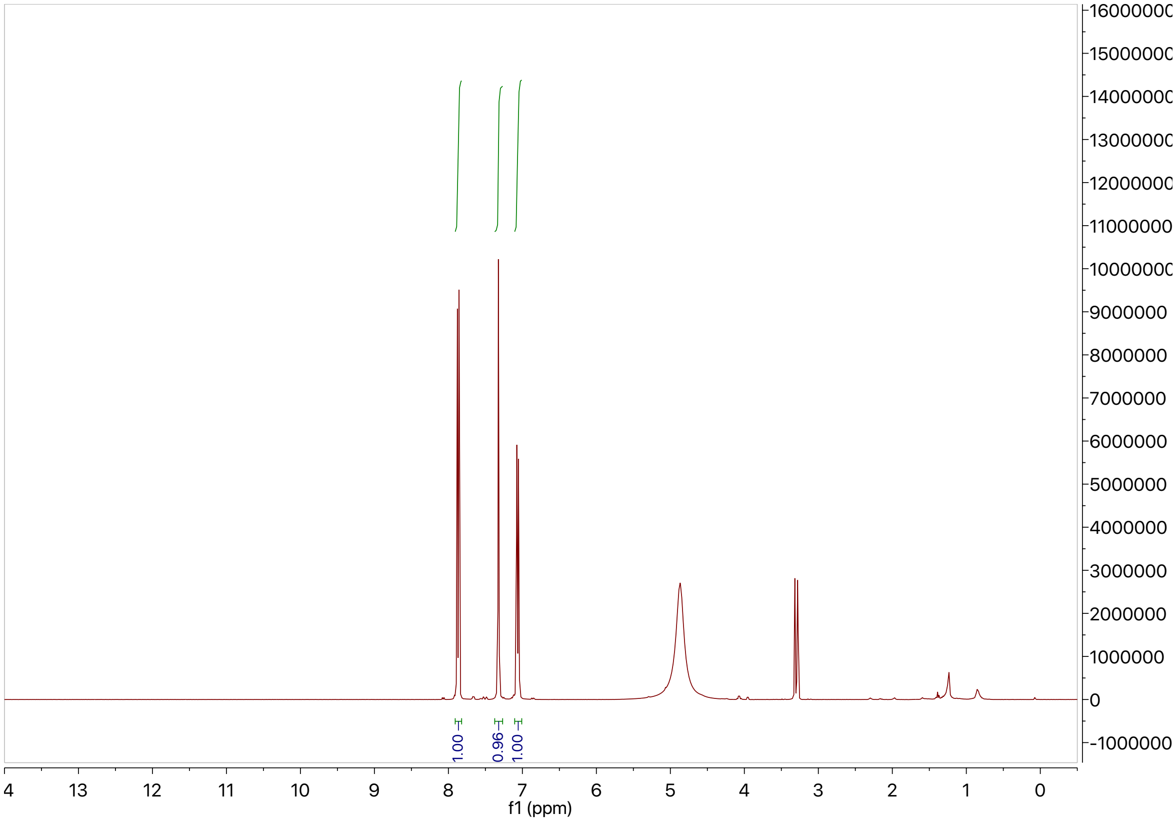

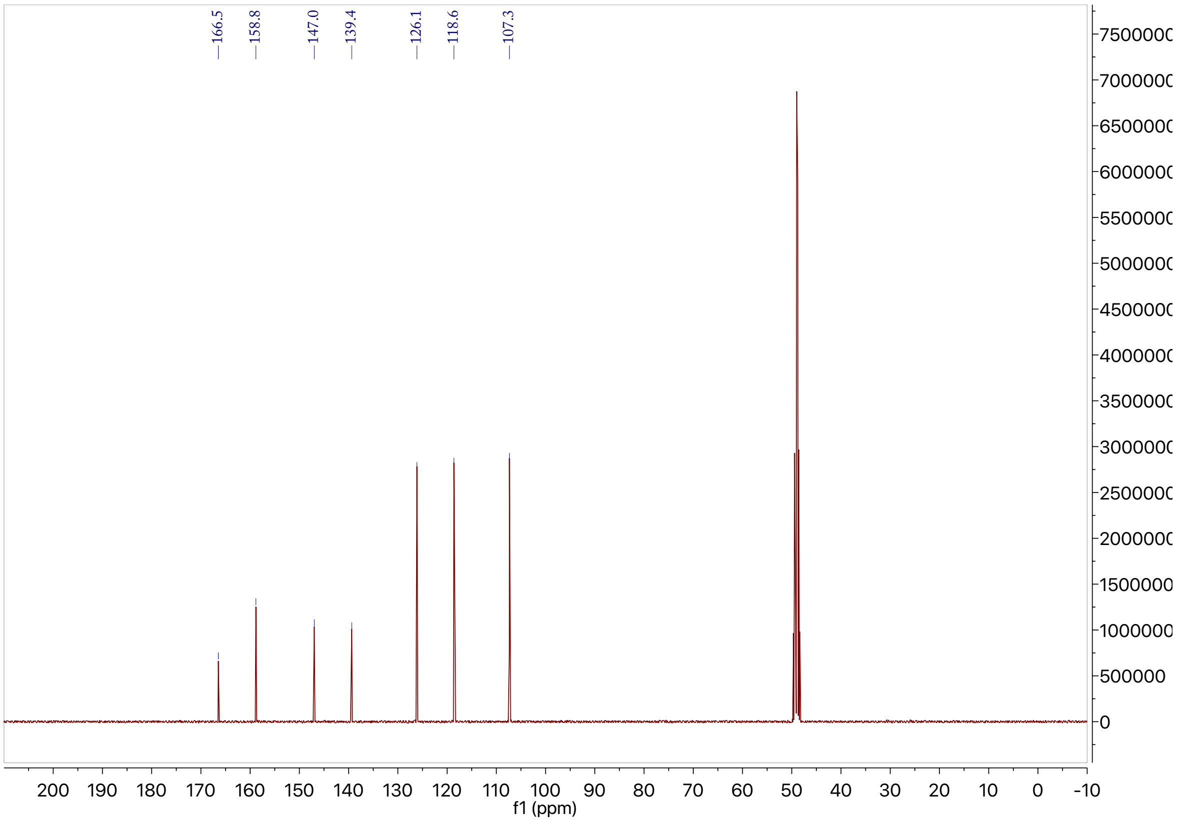

166.5
158.8
147.0
139.4
126.1
118.6
107.3
7500000
7000000
6500000
6000000
5500000
5000000
4500000
4000000
3500000
3000000
2500000
2000000
1500000
1000000
500000
0
200 190 180 170 160 150 140 130 120 110 100 90 80 70 60 50 40 30 20 10 0 -10
f1 (ppm)

Compound 5:

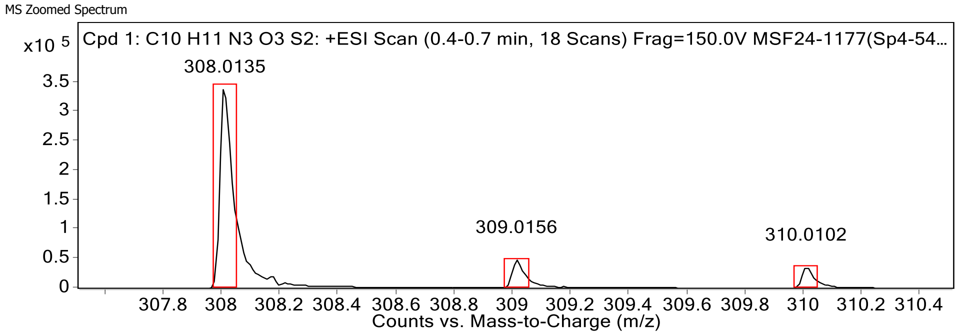

MS Spectrum Peak List

Obs. m/z	Calc. m/z	Charge	Abundance	Formula	Ion Species	Tgt Mass Error (ppm)
308.0135	308.0134	1	346327	C10H11N3O3S2	(M+Na)+	-0.39
309.0156	309.0158	1	48026	C10H11N3O3S2	(M+Na)+	0.87
310.0102	310.0105	1	35181	C10H11N3O3S2	(M+Na)+	0.8
311.0134	311.0126	1	4420	C10H11N3O3S2	(M+Na)+	-2.59
593.0381			559307			

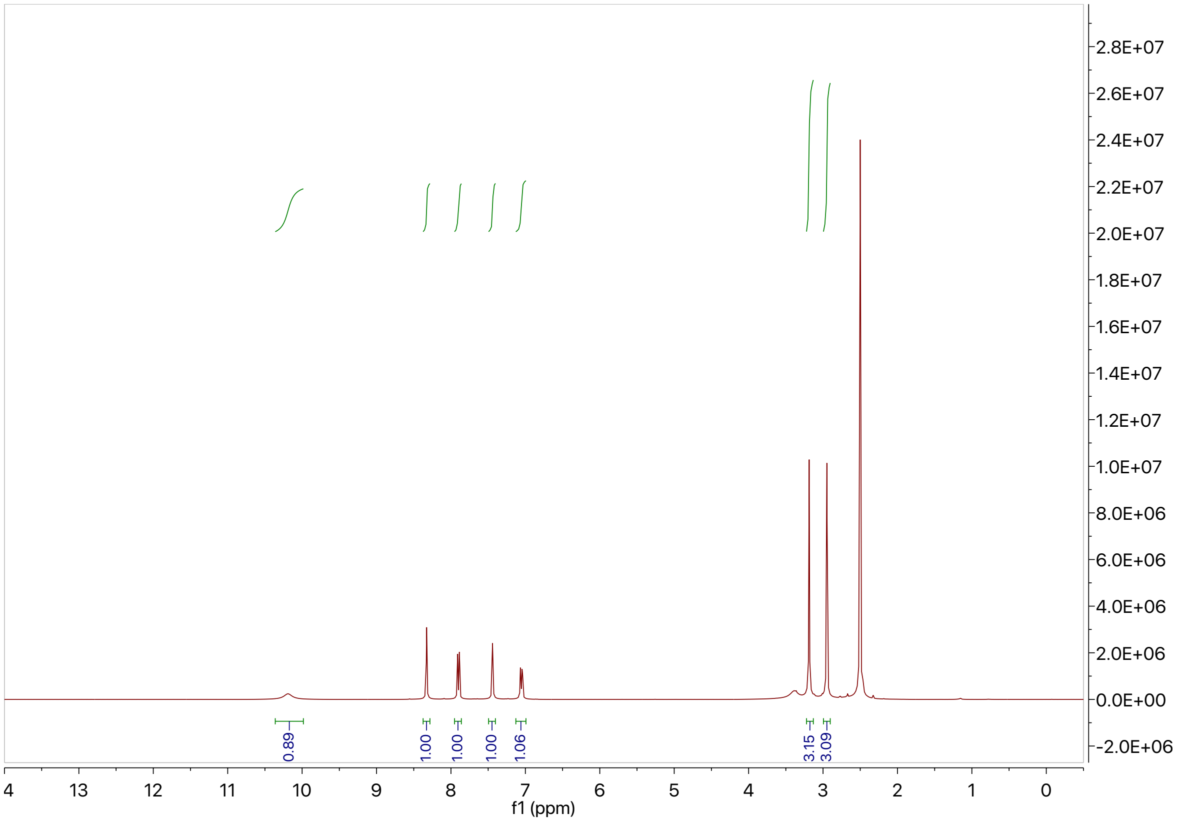

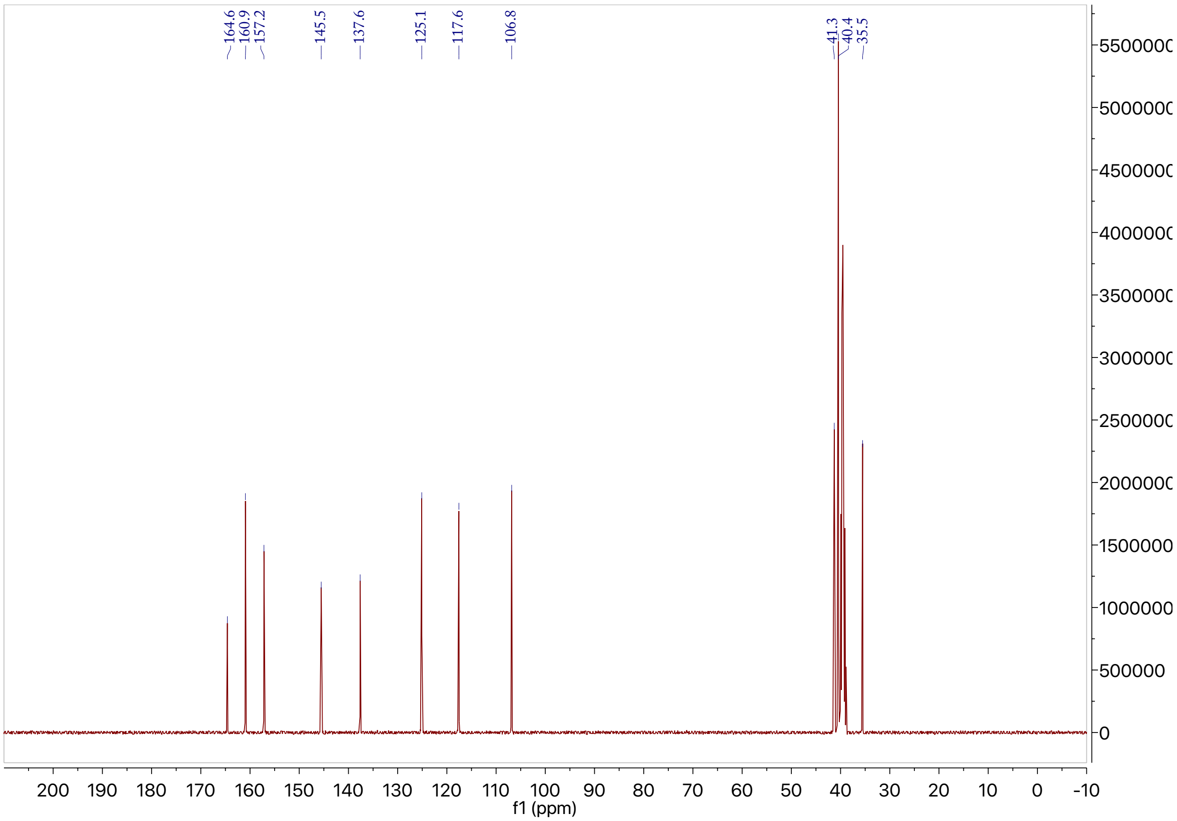
164.6
160.9
157.2
145.5
137.6
125.1
117.6
106.8
41.3
40.4
35.5
5500000
5000000
4500000
4000000
3500000
3000000
2500000
2000000
1500000
1000000
500000
0
200 190 180 170 160 150 140 130 120 110 100 90 80 70 60 50 40 30 20 10 0 -10
f1 (ppm)

Compound 6:

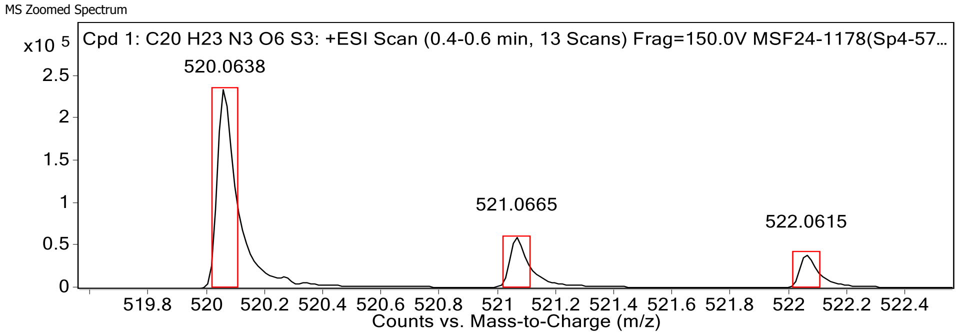

MS Spectrum Peak List

Obs. m/z	Calc. m/z	Charge	Abundance	Formula	Ion Species	Tgt Mass Error (ppm)
520.0638	520.0641	1	236173	C20H23N3O6S3	(M+Na)+	0.66
521.0665	521.0669	1	59987	C20H23N3O6S3	(M+Na)+	0.76
522.0615	522.0622	1	39806	C20H23N3O6S3	(M+Na)+	1.41
523.0630	523.0641	1	9465	C20H23N3O6S3	(M+Na)+	1.98
524.0618	524.0609	1	2715	C20H23N3O6S3	(M+Na)+	-1.65

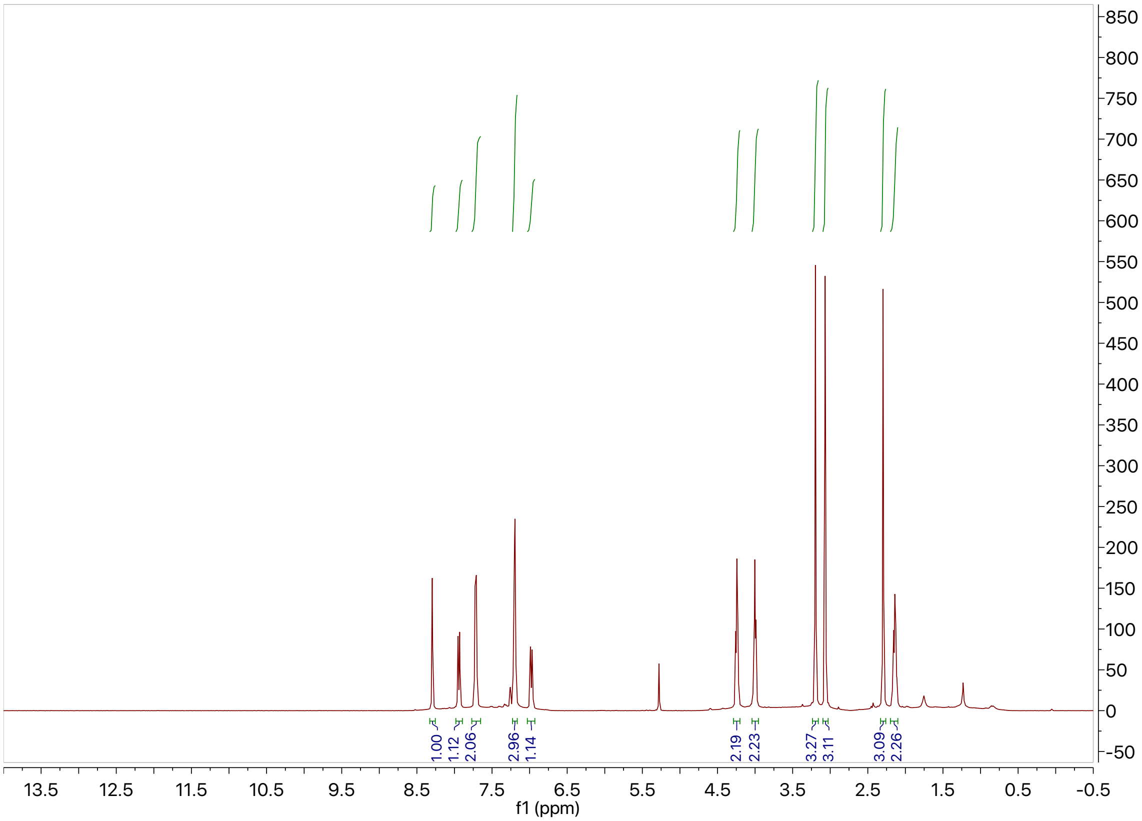

\

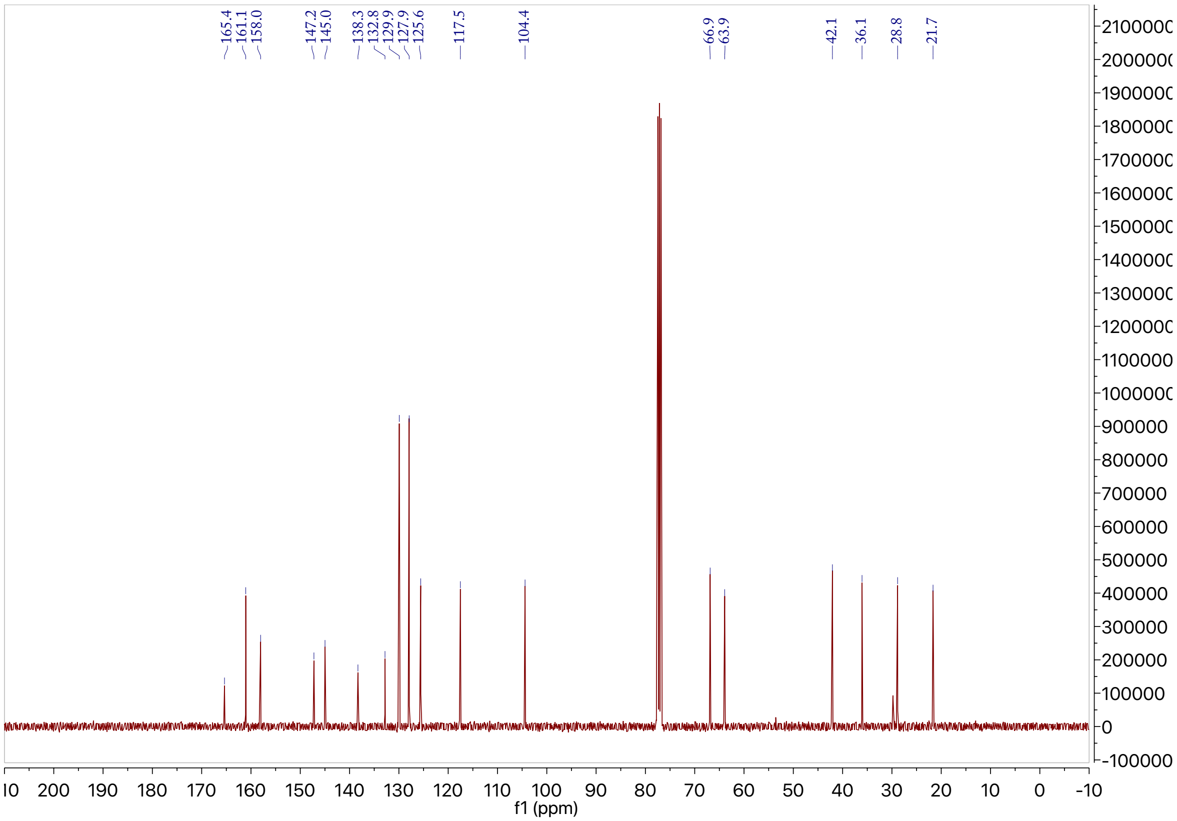

165.4
161.1
158.0
147.2
145.0
138.3
132.8
129.9
127.9
125.6
117.5
104.4
66.9
63.9
42.1
36.1
28.8
21.7
f1 (ppm)

Compound 7:

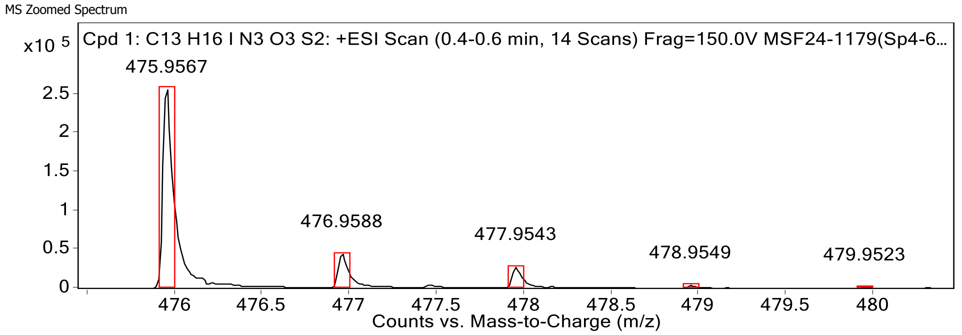

MS Spectrum Peak List

Obs. m/z	Calc. m/z	Charge	Abundance	Formula	Ion Species	Tgt Mass Error (ppm)
475.9567	475.9570	1	258968	C13H16IN3O3S2	(M+Na)+	0.65
476.9588	476.9596	1	44087	C13H16IN3O3S2	(M+Na)+	1.64
477.9543	477.9544	1	27611	C13H16IN3O3S2	(M+Na)+	0.33
478.9549	478.9565	1	3915	C13H16IN3O3S2	(M+Na)+	3.29
479.9523	479.9532	1	1235	C13H16IN3O3S2	(M+Na)+	1.86

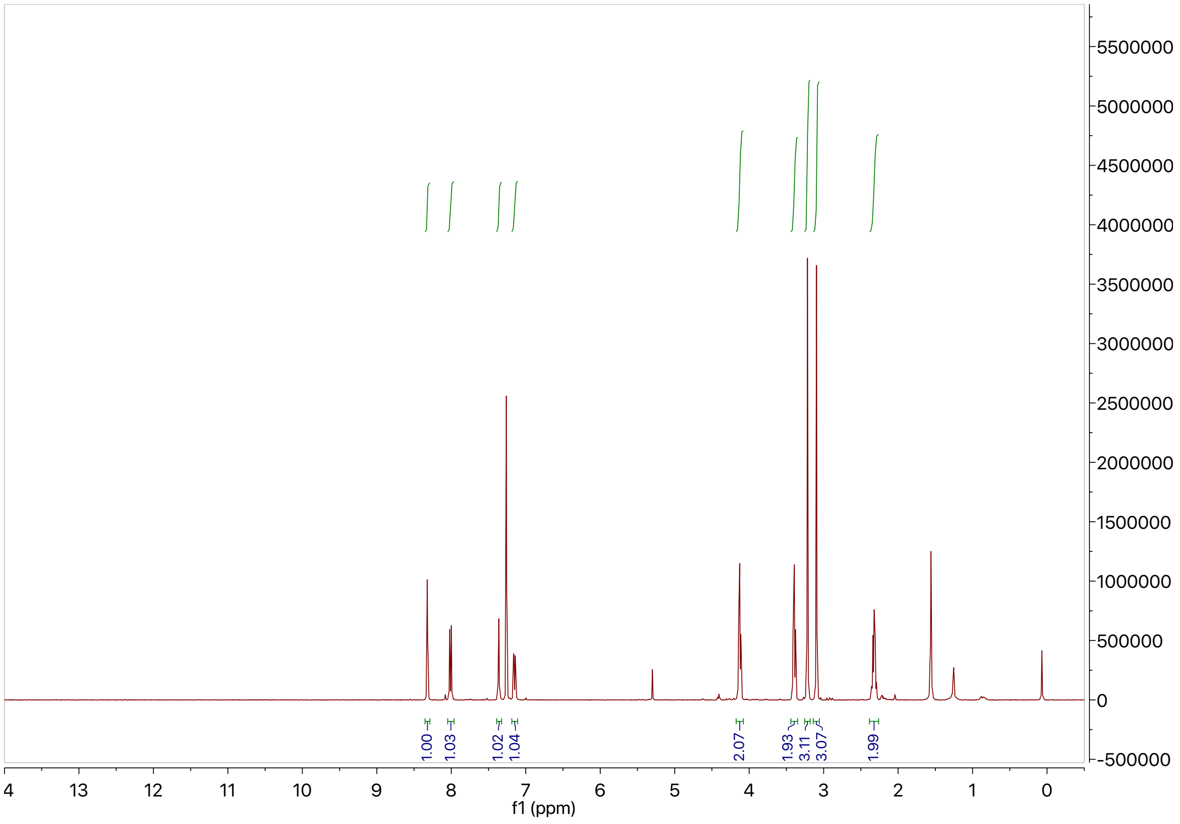

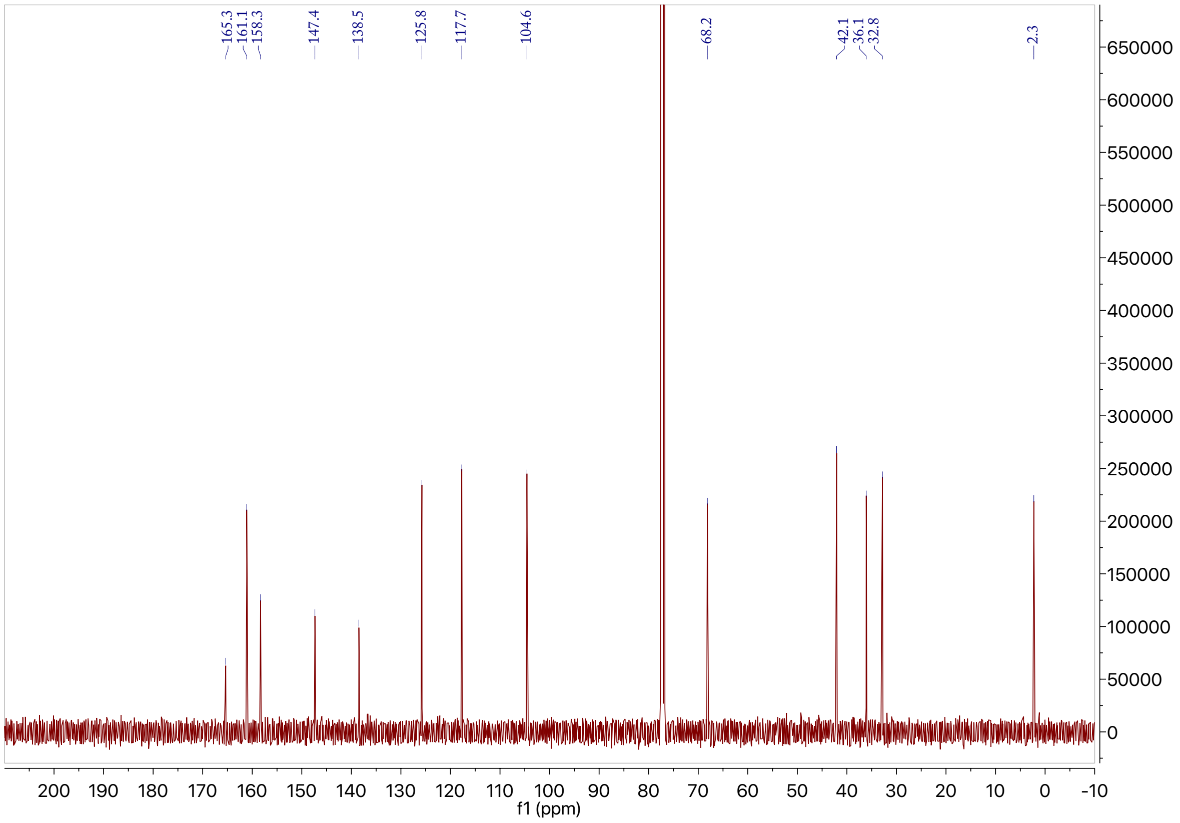
165.3
161.1
158.3
147.4
138.5
125.8
117.7
104.6
68.2
42.1
36.1
32.8
2.3
650000
600000
550000
500000
450000
400000
350000
300000
250000
200000
150000
100000
50000
0
200 190 180 170 160 150 140 130 120 110 100 90 80 70 60 50 40 30 20 10 0 -10
f1 (ppm)

Compound 8:

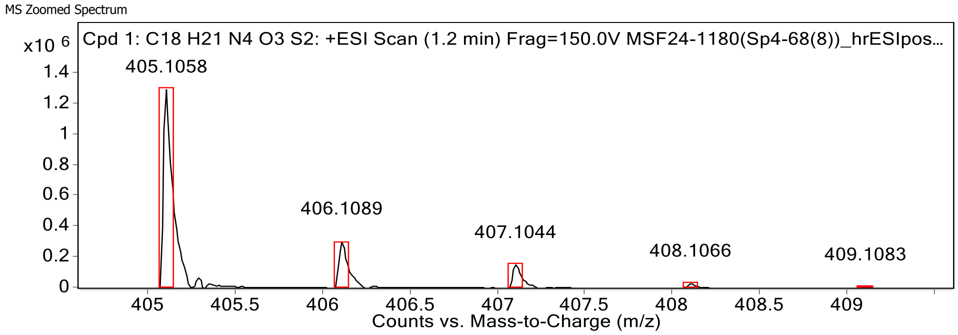

MS Spectrum Peak List

Obs. m/z	Calc. m/z	Charge	Abundance	Formula	Ion Species	Tgt Mass Error (ppm)
405.1058	405.1050	1	1294486	C18H21N4O3S2	M+	-2.16
406.1089	406.1077	1	305000	C18H21N4O3S2	M+	-3.08
407.1044	407.1032	1	151177	C18H21N4O3S2	M+	-2.9
408.1066	408.1048	1	30314	C18H21N4O3S2	M+	-4.39
409.1083	409.1025	1	6427	C18H21N4O3S2	M+	-13.98

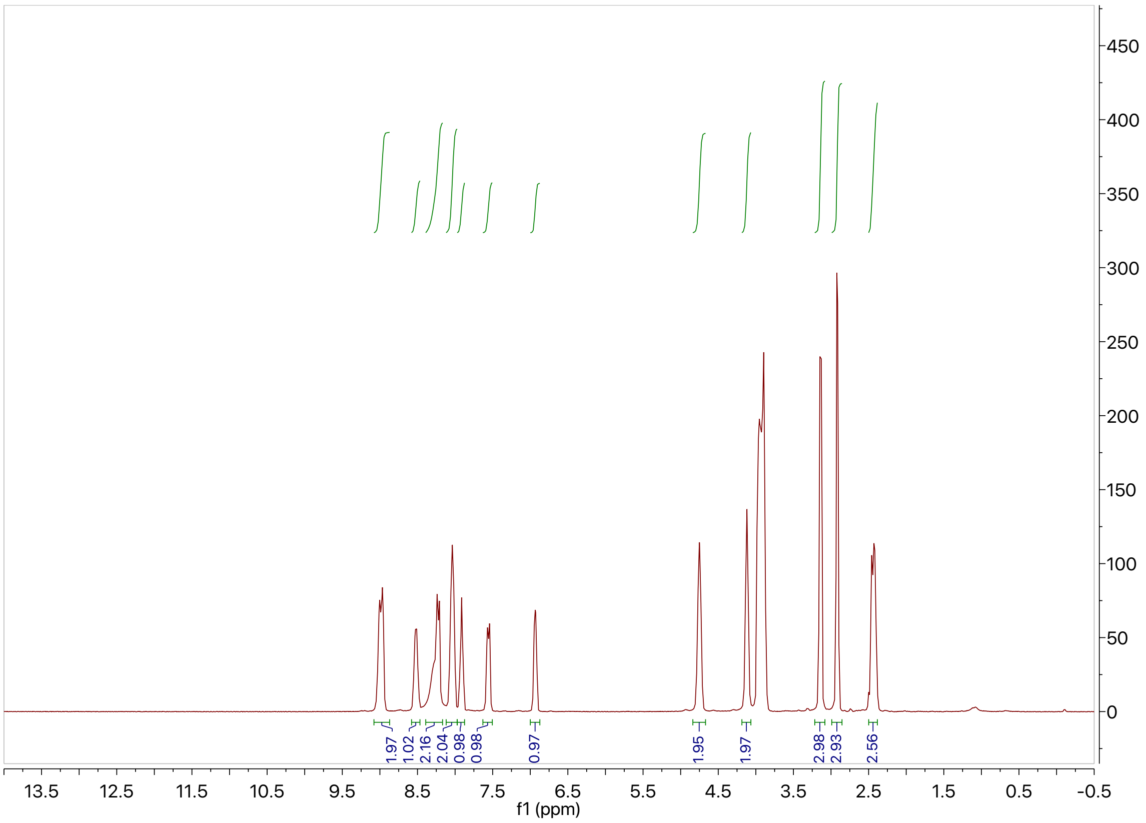

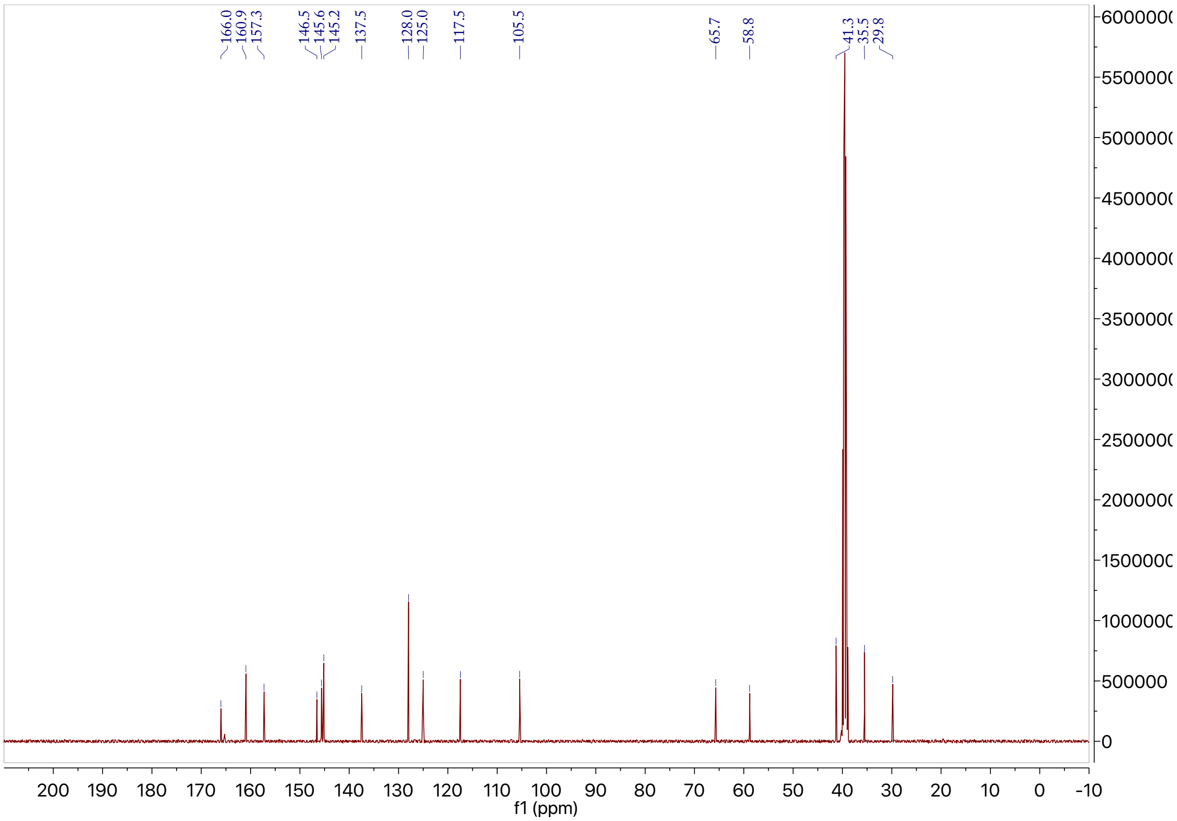
166.0
160.9
157.3
146.5
145.6
145.2
137.5
128.0
125.0
117.5
105.5
65.7
58.8
41.3
35.5
29.8
f1 (ppm)

Compound 9:

MS Zoomed Spectrum

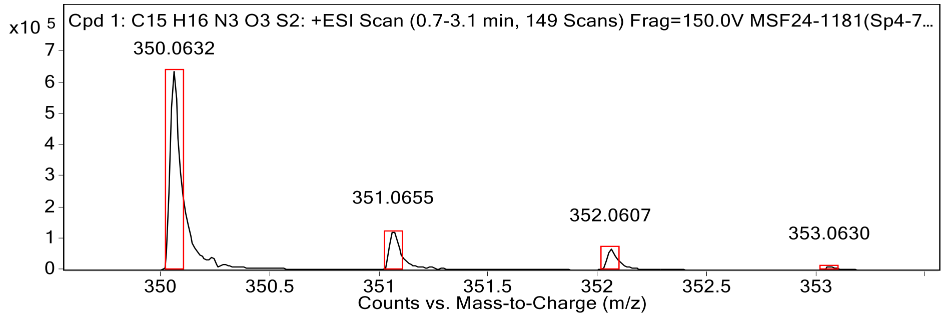

MS Spectrum Peak List

Obs. m/z	Calc. m/z	Charge	Abundance	Formula	Ion Species	Tgt Mass Error (ppm)
350.0632	350.0628	1	637501	C15H16N3O3S2	M+	-1.16
351.0655	351.0655	1	126587	C15H16N3O3S2	M+	-0.07
352.0607	352.0605	1	70067	C15H16N3O3S2	M+	-0.46
353.0630	353.0624	1	11981	C15H16N3O3S2	M+	-1.67

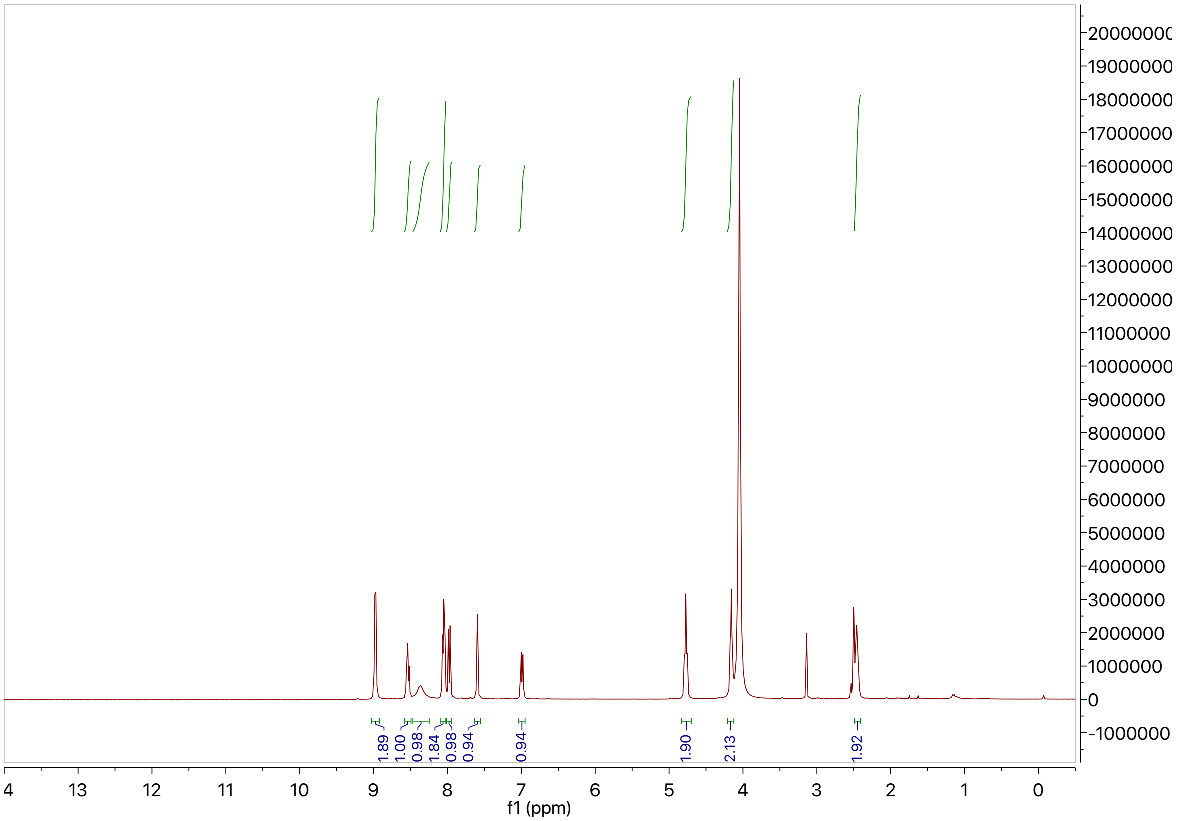

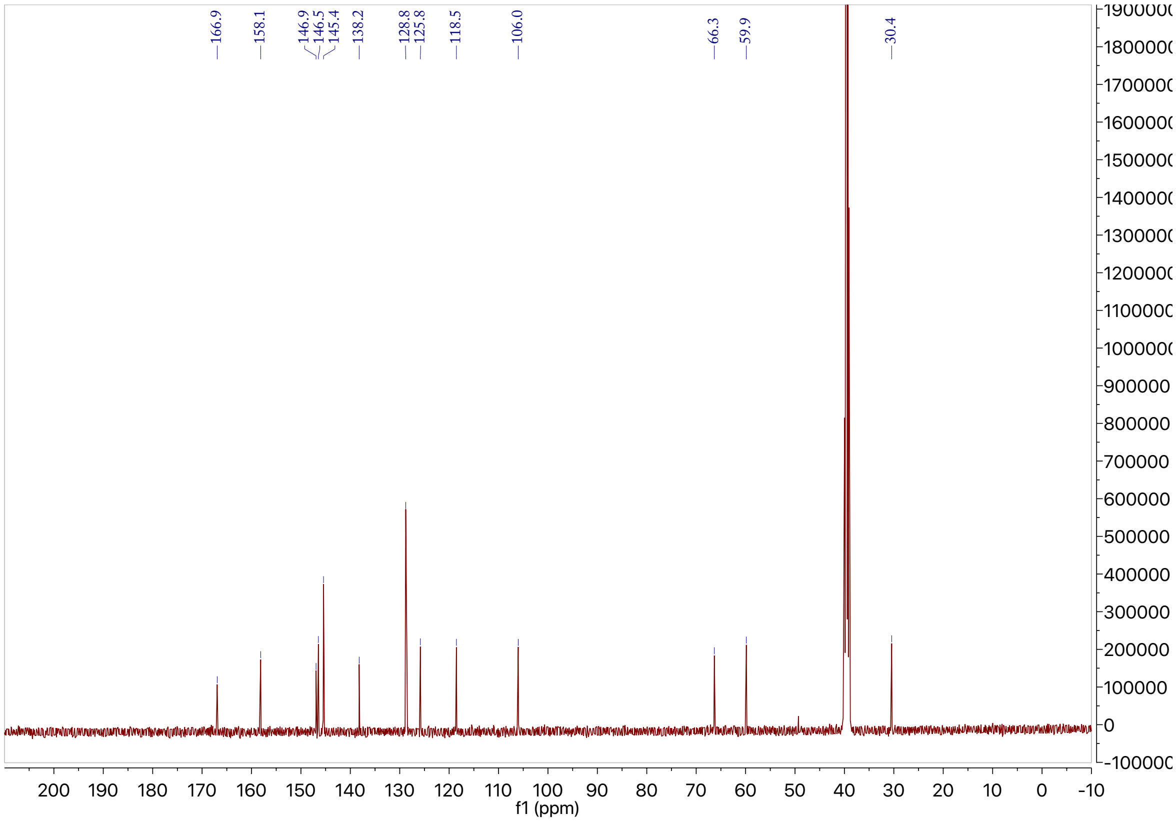

166.9
158.1
146.9
146.5
145.4
138.2
128.8
125.8
118.5
106.0
66.3
59.9
30.4
200 190 180 170 160 150 140 130 120 110 100 90 80 70 60 50 40 30 20 10 0 -10
f1 (ppm)

PCE:

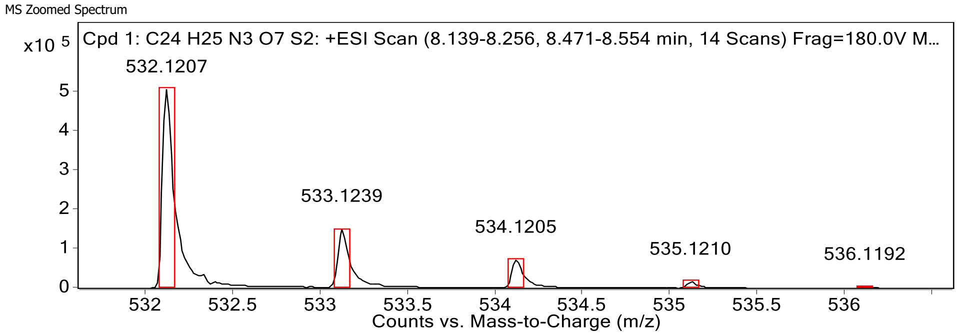

MS Spectrum Peak List

Obs. m/z	Calc. m/z	Charge	Abundance	Formula	Ion Species	Tgt Mass Error (ppm)
532.1207	532.1207	1	507930	C24H25N3O7S2	(M+H)+	-0.09
533.1239	533.1236	1	152147	C24H25N3O7S2	(M+H)+	-0.49
534.1205	534.1202	1	73105	C24H25N3O7S2	(M+H)+	-0.65
535.1210	535.1217	1	16960	C24H25N3O7S2	(M+H)+	1.17
536.1192	536.1204	1	3706	C24H25N3O7S2	(M+H)+	2.4

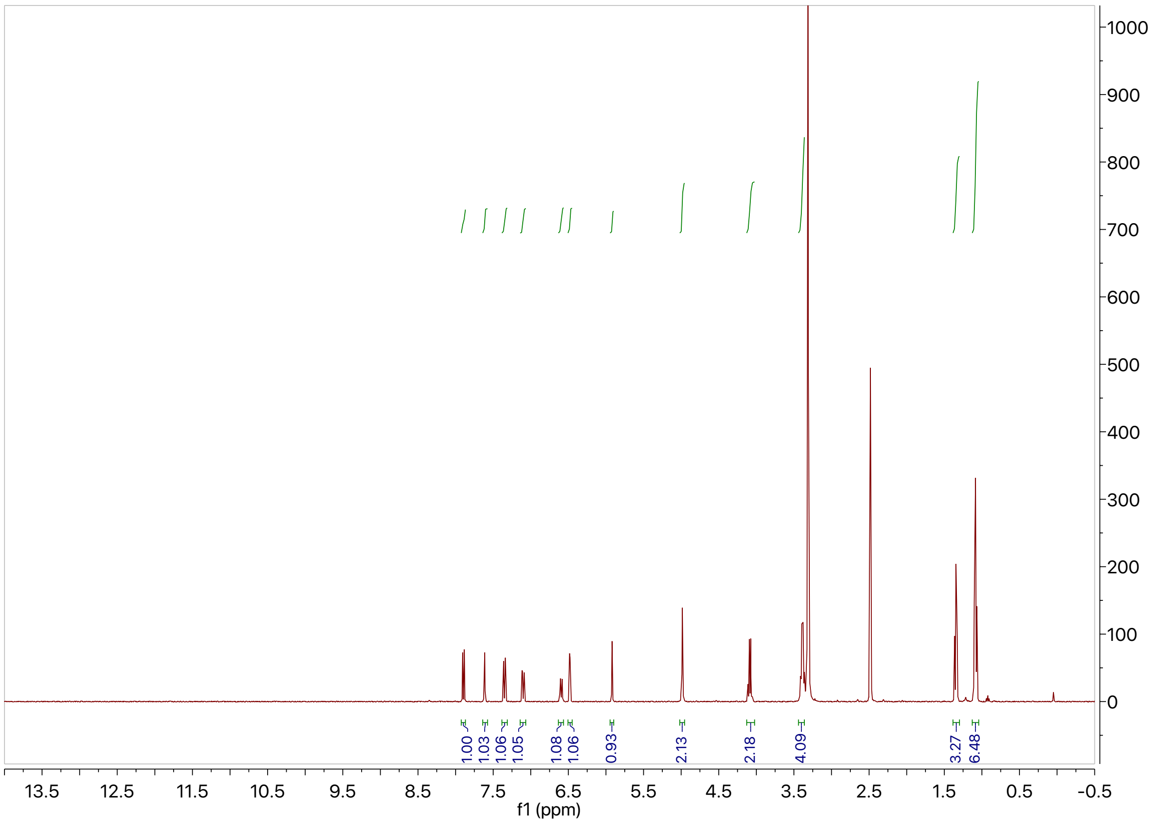

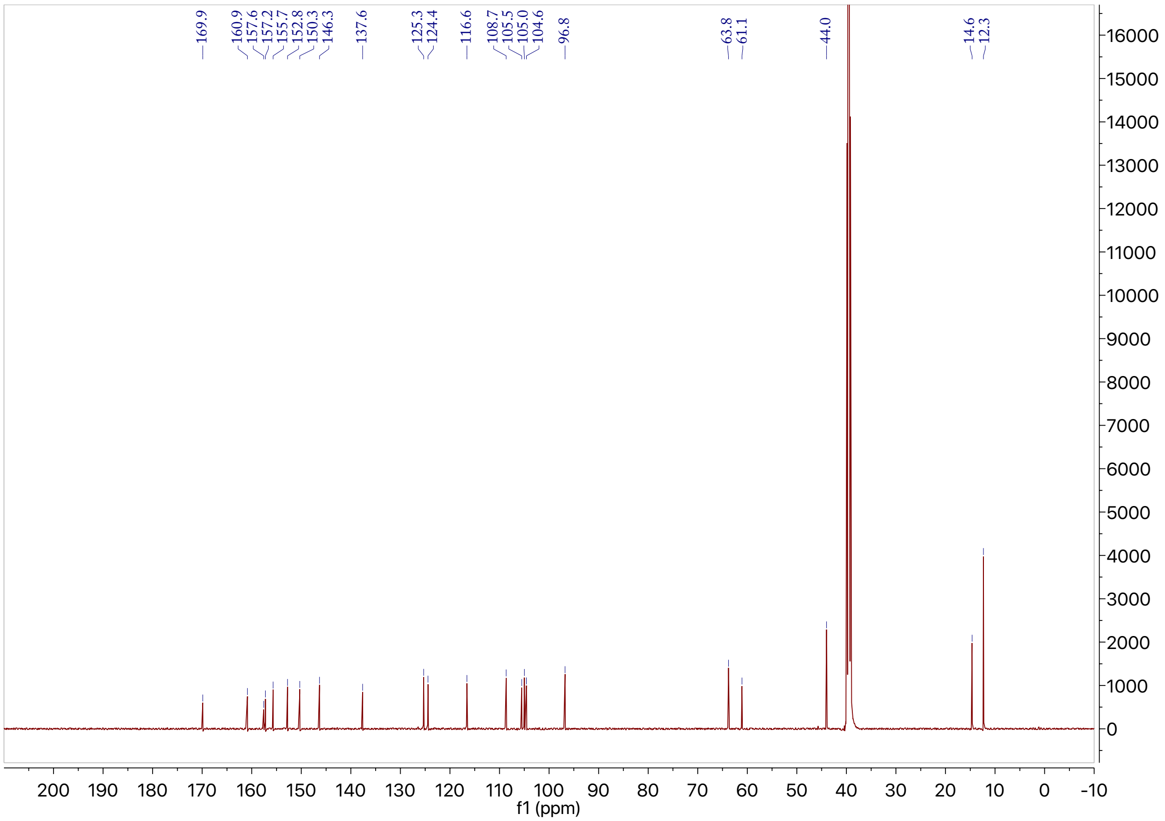

169.9
160.9
157.6
157.2
155.7
152.8
150.3
146.3
137.6
125.3
124.4
116.6
108.7
105.5
105.0
104.6
96.8
63.8
61.1
44.0
14.6
12.3
16000
15000
14000
13000
12000
11000
10000
9000
8000
7000
6000
5000
4000
3000
2000
1000
0
200 190 180 170 160 150 140 130 120 110 100 90 80 70 60 50 40 30 20 10 0 -10
f1 (ppm)

PCEM:

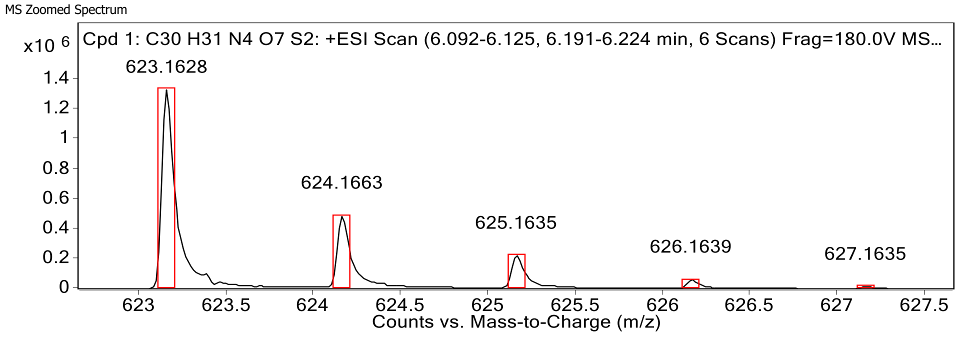

MS Spectrum Peak List

Obs. m/z	Calc. m/z	Charge	Abundance	Formula	Ion Species	Tgt Mass Error (ppm)
623.1628	623.1629	1	1333494	C30H31N4O7S2	M+	0.12
624.1663	624.1658	1	489620	C30H31N4O7S2	M+	-0.77
625.1635	625.1632	1	221112	C30H31N4O7S2	M+	-0.48
626.1639	626.1643	1	61136	C30H31N4O7S2	M+	0.62
627.1635	627.1638	1	13022	C30H31N4O7S2	M+	0.36

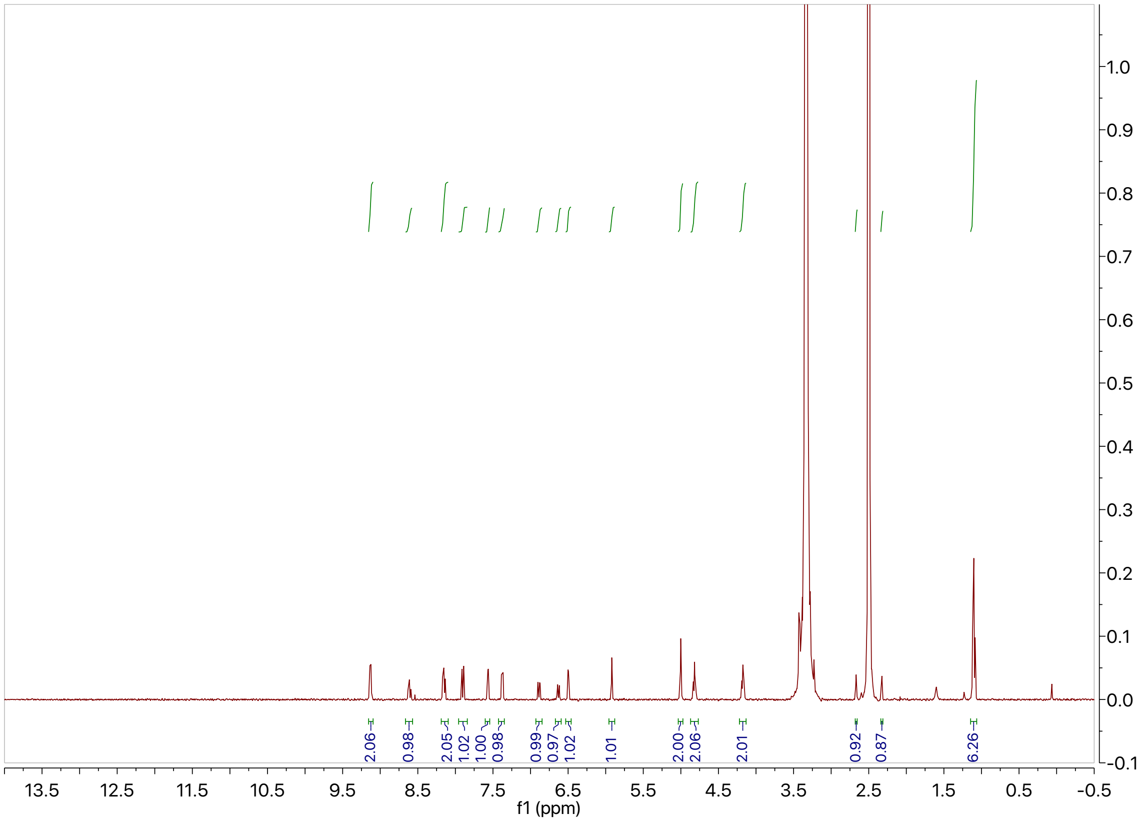

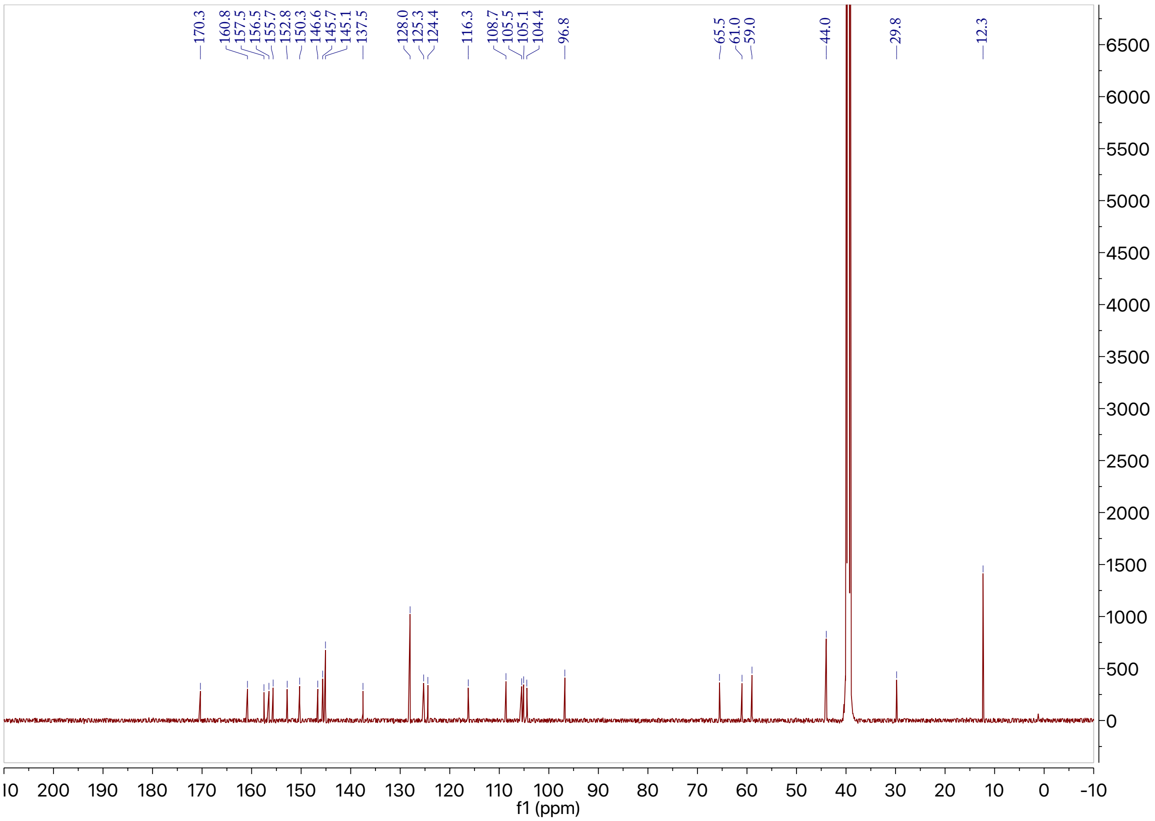
170.3
160.8
157.5
156.5
155.7
152.8
150.3
146.6
145.7
145.1
137.5
128.0
125.3
124.4
116.3
108.7
105.5
105.1
104.4
96.8
65.5
61.0
59.0
44.0
29.8
12.3
f1 (ppm)
